# Role of Suprabasin in the Dedifferentiation of Follicular Epithelial Cell-Derived Thyroid Cancer and Identification of Related Immune Markers

**DOI:** 10.3389/fgene.2022.810681

**Published:** 2022-02-09

**Authors:** Hao Tan, Lidong Wang, Zhen Liu

**Affiliations:** Department of General Surgery, Shengjing Hospital of China Medical University, Shenyang, China

**Keywords:** suprabasin, thyroid cancer, lymph node metastasis, immune infiltration, tumor immunosuppression, dedifferentiation

## Abstract

**Background:** Aberrant regulation of suprabasin (SBSN) is associated with the development of cancer and immune disorders. SBSN influences tumor cell migration, proliferation, angiogenesis, and immune resistance. In this study, we investigated the potential correlation between *SBSN* expression and immune infiltration in thyroid cancer.

**Methods:** The expression of SBSN in 80 papillary thyroid carcinoma (PTC) specimens was determined using quantitative reverse-transcription polymerase chain reaction, western blotting, and immunohistochemical staining. The expression of SBSN in 9 cases of poorly differentiated thyroid carcinoma (PDTC) and 18 cases of anaplastic thyroid carcinoma (ATC) was evaluated by immunohistochemical staining. Comprehensive bioinformatics analysis of *SBSN* expression was performed using The *Cancer* Genome Atlas and Gene Expression Omnibus datasets, and the relationship of SBSN expression with M2 macrophages and T regulatory cells (Tregs) in ATC and PTC was verified by immunohistochemical staining.

**Results:** Compared with those in adjacent normal tissues, the expression levels of SBSN mRNA and protein were significantly higher in PTC tissues. SBSN expression level was correlated with that of cervical lymph node metastasis in PTC patients. Immunohistochemical staining results showed statistically significant differences among high-positive expression rates of SBSN in PTC, PDTC, and ATC. Functional enrichment analysis showed that *SBSN* expression was associated with pathways related to cancer, cell signaling, and immune response. Furthermore, analysis of the tumor microenvironment (using CIBERSORT-ABS and xCell algorithms) showed that *SBSN* expression affected immune cell infiltration and the cancer immunity cycle, and immunohistochemistry confirmed a significant increase in M2 macrophage and Treg infiltration in tumor tissues with high-positive SBSN expression.

**Conclusion:** These findings reveal that SBSN may be involved in thyroid carcinogenesis, tumor dedifferentiation progression, and immunosuppression as an important regulator of tumor immune cell infiltration.

## Introduction

Thyroid cancer is one of the most common endocrine tumors and its incidence has increased globally over the last 30 years (La Vecchia et al., 2015). Most thyroid cancers originate from the follicular epithelial cells of the thyroid gland, which secrete iodine-containing thyroid hormones. Follicular epithelium-derived thyroid cancers can be classified as papillary thyroid carcinoma (PTC), follicular thyroid carcinoma, poorly differentiated thyroid carcinoma (PDTC), and anaplastic thyroid carcinoma (ATC) (Dralle et al., 2015). Of these, PTC is the most common pathological type, accounting for 80–90% of all thyroid cancers (Abdullah et al., 2019). Most thyroid cancers exhibit inert biological behavior and have a good prognosis, with a 20-year survival rate of 95% (Gospodarowicz et al., 2001). However, recurrence, metastasis, and resistance to radioiodine therapy in PDTC, ATC, and some invasive PTCs remain the leading causes of death from thyroid cancer (Mazzaferri and Jhiang, 1994; Molinaro et al., 2017; Xu and Ghossein, 2020), with more than 25% of patients with PTC experiencing recurrence during a long-term follow-up (Abdullah et al., 2019). Furthermore, a high rate (up to 85%) of cervical-lymph-node metastasis, which is considered a very high-risk factor for PTC recurrence, has been documented in patients with PTC (Zheng et al., 2019). Currently, some studies suggest that ATC is different from PTC in the early stages of tumor development and that the two tumor types evolve via distinct mechanisms (Capdevila et al., 2018). However, it is also believed that histologically highly differentiated thyroid cancer may dedifferentiate into PDTC or ATC via a multi-step process of genetic and epigenetic alterations (Papp and Asa, 2015) or that ATC can develop from PTC via accumulation of genomic mutations (Landa et al., 2016).

Immunotherapy has long been a focus area in oncology and is effective against non-small cell lung cancer (NSCLC) and kidney cancer (Motzer et al., 2015; Reck et al., 2016). Immune cell infiltration of the tumor microenvironment (TME) has also been associated with survival in many patients with solid tumors (Baxevanis et al., 2019). Infiltrating immune cells may be used as drug targets to improve patient survival (Lote et al., 2015). A previous study showed that the polarization of a higher number of tumor-associated macrophages (TAMs) in a tumor-promoting M2 phenotype implies a poorer survival of patients with ATC (Jung et al., 2015). Fang et al. (2014) found that TAMs purified from human PTC could promote invasiveness of thyroid cancer cell lines by secreting CXCL8. Melillo et al. (2010) found that the density of tumor-associated mast cells was higher in PTC than in normal tissue and correlated with extra-thyroidal tumor infiltration. In PTC, the number of CD4^+^ T cells correlates with the tumor size, whereas that of Tregs correlates with lymph node metastasis (French et al., 2010). Tregs are enriched in tumor-involved lymph nodes, and their numbers correlate with PTC recurrence (French et al., 2012). Tumor-infiltrating lymphocytes, TAMs, and tumor-infiltrating neutrophils influence the prognosis and efficacy of chemotherapy and immunotherapy (Waniczek et al., 2017; Zhang et al., 2018). In addition, Chen and Mellman (2013) divided the cancer immunity cycle into seven steps, including the release of cancer cell antigens, cancer antigen presentation, initiation and activation of immune cells, transport and infiltration of immune cells into the tumor, and recognition and killing of cancer cells by T cells. Consequently, the cancer immunity cycle has become one of the starting points for cancer immunotherapy research. Therefore, it is imperative to study the TME and identify the distribution and functions of tumor-infiltrating immune cells (TIICs) to find new tumor markers for thyroid cancer.

SBSN was first identified in epithelial tissues (human and murine) and is thought to play a key role in the process of epidermal differentiation (Park et al., 2002). However, in recent years, SBSN has been reported to be aberrantly expressed in certain malignancies, and inhibition of SBSN may lead to the inhibition of cancer cell proliferation, invasion, and metastasis, suggesting that SBSN may be associated with tumor progression. For example, SBSN expression is abnormally regulated in esophageal squamous cell carcinoma (ESCC) (Zhu et al., 2016; Takahashi et al., 2020), salivary adenoid cystic carcinoma (ACC) (Shao et al., 2012), and NSCLC (Glazer et al., 2009). In addition, several studies have shown that SBSN expression in tumors is regulated by several signaling pathways that affect the tumor properties (Alam et al., 2014; Zhu et al., 2016; Takahashi et al., 2020). These results suggest that SBSN may act as an oncogenic factor to promote tumorigenesis and tumor progression. In addition, SBSN plays an important role in the development and progression of immune diseases, such as neuropsychiatric systemic lupus erythematosus (Ichinose et al., 2018) and atopic dermatitis (Aoshima et al., 2019). However, the potential mechanism of *SBSN* as a proto-oncogene in thyroid cancer progression and immunology is not clear.

In this study, we investigated the expression of SBSN in thyroid cancers of follicular epithelial origin with different degrees of differentiation. The relationship of SBSN with clinicopathological features of patients with PTC, as well as the potential involvement of SBSN in cancer immunity, were also explored. This study suggests an important role for SBSN in thyroid carcinoma and the potential mechanisms by which SBSN may be involved in the processes of thyroid cancer dedifferentiation and immune regulation.

## Materials and Methods

### Data Sources and Pre-Processing

This study used several public datasets, including PTC, PDTC, and ATC datasets. For The *Cancer* Genome Atlas (TCGA) dataset, RNA sequencing (RNA-seq) data and clinical features of patients with thyroid cancer were identified in and extracted from the TCGA portal and validated (Cancer Genome Atlas Research Network, 2014), with a total of 568 samples, including 502 PTCs, 8 metastatic thyroid cancers, and 58 matched normal thyroid samples. High-throughput sequencing fragments per kilobase of transcript per million mapped reads (FPKM) values were further analyzed for all samples after log_2_(FPKM+1) transformation. The ATC microarray datasets GSE29265, GSE33630, GSE76039, and GSE65144 were downloaded from the Gene Expression Omnibus database of the National Center for Biotechnology Information by searching for “anaplastic thyroid cancer” and “*Homo sapiens*.” The PDTC microarray datasets GSE53157 and GSE76039 were downloaded from the same database by searching for “poorly differentiated thyroid cancer” and “*Homo sapiens*.” All microarray data were background adjusted and normalized by removing the batch processing effect using the R package “sva.” Probes that did not match the gene symbol in the annotation file were deleted. When more than one probe matched the same gene symbol, the average value was calculated as the final expression value. GSE29265 consisted of 9 ATC tissues and 10 adjacent normal thyroid tissues. GSE33630 consisted of 11 ATC tissues and 45 adjacent normal thyroid tissues. GSE65144 consisted of 12 ATC tissues and 13 adjacent normal thyroid tissues. GSE76039 consisted of 17 PDTC tissues and 20 ATC tissues. GSE53157 consisted of five PDTCs, seven classical PTCs, eight PTC follicular variants, and four follicular thyroid carcinomas.

### Immune Infiltration Analysis

The ESTIMATE algorithm can determine the ratio of stromal and immune cells based on the gene expression profile in tumor samples. It has been applied to assess the TME in patients, as well as the stromal score (stromal cell content), immune score (degree of immune cell infiltration), ESTIMATE score (a synthetic marker of the stroma and immunity), and tumor purity, using the R package (Yoshihara et al., 2013). The CIBERSORT-ABS and xCell algorithms were used to estimate the relative proportions of various immune cell types in the TME. For each cell type, xCell was used to analyze the enrichment scores of all samples by integrating a single-sample gene set enrichment analysis (ssGSEA) approach (Aran et al., 2017). CIBERSORT-ABS, an analytical method developed by Newman, uses gene expression data to estimate the abundance ratios of 22 cell types in a mixed cell population at a statistical significance level of *p* < 0.05 (Newman et al., 2015). The reference for the deconvolution of RNA-seq data from patients with cancer was the leukocyte signature matrix (LM22). For cancer immunity cycle analysis, we applied the Tracking Tumor Immunophenotype (TIP) pipeline based on the ssGSEA algorithm (Xu et al., 2018). The TIP scores for the TCGA dataset are available from the online TIP server (http://biocc.hrbmu.edu.cn/TIP/).

### Patients and Clinicopathological Data

Eighty specimens from patients with PTC who underwent surgical treatment at the Shengjing Hospital of China Medical University from July 2016 to July 2017 were stored in liquid nitrogen and subjected to quantitative reverse-transcription polymerase chain reaction (qRT-PCR) and western blotting. For immunohistochemical staining, tissues were embedded in paraffin. The selected PTC tissues and paired normal tissues adjacent to cancer were diagnosed by pathology. Normal tissues adjacent to the cancer tissue were collected, from the same patients with PTC, at least 2 cm from the PTC area. Paraffin-embedded tissues from 9 patients with PDTC and 18 patients with ATC who underwent surgical treatment at the Shengjing Hospital of China Medical University from 2010 to 2017 were preserved at the Department of Pathology. All histological sections were reviewed by two specialist pathologists to verify the histological diagnosis. All patients were diagnosed for the first time and did not receive any treatment before surgery. Patients were classified according to the eighth edition of the American Joint Committee on *Cancer* TNM classification system for differentiated thyroid cancer. Clinical information such as patient age, tumor size, and cervical-lymph-node metastasis was retrieved from the clinical files of the patients. All patients provided informed consent for the use of their clinical and pathological data for research purposes, and all tissue specimens and clinical data were collected according to the protocol approved by the Ethics Committee of the Shengjing Hospital, China Medical University (approval number 2014PS47K).

### Functional and Pathway Enrichment Analyses

The GeneMANIA (http://www.genemania.org) database was used to construct a gene-gene interaction network for *SBSN*, including genes that are associated with SBSN in terms of physical interactions, co-expression, prediction, co-localization, and genetic interactions. Functional and pathway enrichment analyses of the genes co-expressed with *SBSN* in the cBioPortal database (496 cases from the TCGA Cell 2014 dataset of PTC) were performed using DAVID (https://david.ncifcrf.gov). Genome enrichment analysis was performed using GSEA 3.0 software. The c2.cp.kegg.v6.1.symbols.gmt dataset was downloaded from the Molecular Signatures Database on the GSEA website. Enrichment analysis of *SBSN* ^high^ and *SBSN* ^low^ groups was performed for expression spectrum data and attribute files using the default weighted method. The random classification frequency was set to 1,000.

### Immunohistochemical Staining

Formalin-fixed, paraffin-embedded sections (4 μm thick) were prepared and subjected to ethanol gradient dewaxing and endogenous peroxidase blocking. Antigen retrieval was performed by boiling the slides in citrate buffer (pH 6.0) for 7.5 min, followed by cooling to room temperature. The slides were incubated with a polyclonal rabbit anti-human SBSN antibody (1:250; Cat# abx130453; Abbexa, Cambridge, United Kingdom) at 4°C overnight after 30 min of incubation at 37°C with drops of goat blocking serum. Afterward, the slides were rinsed with phosphate-buffered saline (PBS) and incubated with a drop of a horseradish peroxidase-labeled sheep anti-rabbit secondary antibody for 30 min at 37°C. Subsequently, the slides were stained with a 3,3′-diaminobenzidine (Cat# DAB-0031; MXB, Maixin, China) solution for 1–2 min, counterstained with hematoxylin, dehydrated, covered with coverslips, and analyzed by light microscopy. PBS was used instead of the primary antibody in a negative control group. The experimental procedure was carried out according to the SP kit instructions. Five high-magnification fields (×400) were randomly selected from each section under a light microscope and scored by two pathologists. The degree of staining was scored from 0 to 4 (0, none; 1, <10%; 2, 10–50%; 3, 51–80%; and 4, >80%). The staining intensity was scored from 0 to 3 (0, no staining; 1, light yellow; 2, brown-yellow; and 3, brown). To calculate the final score, the two scores were multiplied, and the results were presented as follows: 0–1 point (−), 2–4 points (+), 5–8 points (++), and 9–12 points (+++). We defined −/+ as the low-positive expression group and ++/+++ as the high-positive expression group. To control for errors, the scoring was performed by two independent observers, and a third observer read the films; in cases of disagreement, all three discussed the scores collectively until an agreement was reached.

For M2 macrophages, fields of view with CD163^+^ M2 macrophages were selected. The number of CD163^+^ M2 macrophages was counted in five randomized high-magnification fields (×400) per sample, and the mean value was considered as the level of CD163^+^ M2 macrophages.

For Tregs, fields of view with Foxp3^+^ Tregs were selected. The number of Foxp3^+^ Tregs was counted in five randomized high-magnification fields (×400) per sample, and the mean value was considered as the level of Foxp3^+^ Tregs.

### Western Blotting

To extract total proteins, RIPA lysis buffer (Cat#P0013B; Beyotime Biotechnology, Shanghai, China) was added to tissues, and the homogenates were centrifuged at 14,000 × rpm for 45 min at 4°C. Total proteins (40 μg) were separated by SDS-PAGE and transferred to PVDF membranes. The membranes were blocked with 5% bovine serum albumin at room temperature for 2 h and then incubated with primary antibodies at 4°C overnight. Thereafter, the membrane was incubated with a secondary antibody for 2 h at room temperature. The primary antibodies used in this study included a rabbit anti-SBSN polyclonal antibody (1:1,000; Cat# abx130453; Abbexa, Cambridge, United Kingdom) and rabbit polyclonal anti-GAPDH (1:10,000; Cat# 10494-1-AP, Proteintech Group, Inc., Chicago, United States). Peroxidase-labeled goat anti-rabbit or anti-mouse IgG (H + L) (1:2,000; Zhongshan Jinqiao Company, Beijing, China) was used as a secondary antibody. An enhanced chemiluminescence kit (Beyotime Biotechnology) was used for detection. The integrated optical density of each band was measured using Image-Pro Plus 6.0 software (Media Cybernetics Inc., Rockville, MD, United States). The target protein expression level was calculated relative to that of GAPDH, which was used as an internal control.

### qRT-PCR

The total RNA was extracted from thyroid tissue specimens using TRIzol reagent (Cat# 9108; Takara, Beijing, China) according to the manufacturer’s instructions. After verification of the purity and concentration, RNA was reverse transcribed into cDNA using a cDNA synthesis kit (Cat# RR047A; Takara, Beijing, China). qRT-PCR of the cDNA (2 μl per 20 μl reaction) was performed using the TB Green ^®^ Premix Ex Taq TM II kit (Cat# RR820; Takara, Beijing, China) and a 7,500 Fast instrument. Primer sequences for SBSN were forwad:5′-CATGGCGTTAGTCAGGCTGGAAG-3′ and reverse:5′-CCTCCTTGCTGGCTTGGTTGAC-3′. The primer sequences used for GAPDH were forward:5′-GGAGCGAGATCCCTCCAAAAT-3′ and reverse:5′- GGC​TGT​TGT​CAT​ACT​TCT​CAT​GG-3′. The PCR protocol was as follows: 95°C for 2 min, followed by 40 cycles at 95°C for 15 s and 60°C for 30 s. The relative expression level was calculated using the 2^−ΔΔCt^ method using *GAPDH* as a reference gene for normalization.

### Statistical Analysis

Statistical analyses were performed using SPSS (20.0) and R (4.0.3) software. The chi-squared test was used to analyze differences in the degree of SBSN immunohistochemical staining among ATC, PDTC, PTC, and adjacent normal thyroid tissues. The relationships between the statistical results of SBSN immunohistochemical staining and the clinicopathological characteristics of PTC were assessed by the chi-squared test and Fisher’s exact probability test. A *t*-test was used to assess the relationships between the statistical results of SBSN western blotting and qRT-PCR and the clinicopathological characteristics of PTC. Data are expressed as means ± standard deviation. Box plot analysis was performed using the Wilcoxon rank-sum test; correlation between two variables was calculated using Spearman’s rho, and one-way analysis of variance was used for comparison among multiple samples. *p* < 0.05 was considered to be statistically significant.

## Results

### Expression of SBSN in Thyroid Cancer Tissues

**FIGURE 1:**
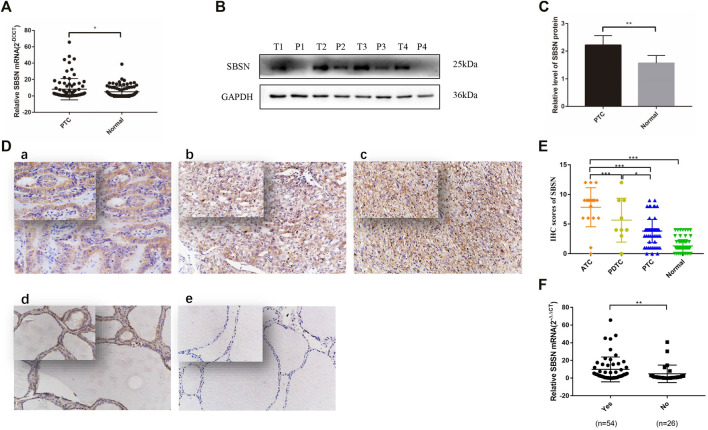
Expression of SBSN in different thyroid tissues. **(A)** Expression of *SBSN* in papillary thyroid carcinoma (PTC) tissues and normal tissues adjacent to cancer was detected by qRT-PCR (*n* = 80 per group). **(B)** Expression of SBSN in PTC and normal tissues adjacent to cancer was detected using western blot (*n* = 80 per group). **(C)** Relative grayscale values of SBSN in PTC tissues and normal tissues adjacent to cancer. **(D)** Expression of SBSN in different thyroid cancer tissue samples (×200, top left ×400). **(a)** Positive expression of SBSN in PTC tissues; **(b)** Positive expression of SBSN in poorly differentiated thyroid carcinoma tissues; **(c)** Positive expression of SBSN in anaplastic thyroid carcinoma tissues; **(d)** Positive expression of SBSN in normal tissues adjacent to cancer; **(e)** Negative expression of SBSN in normal tissues adjacent to cancer. **(E)** Immunohistochemical staining scores of SBSN in various thyroid tissue samples. **(F)** High expression of *SBSN* mRNA levels in PTC tissues was associated with lymph node metastasis in patients. For western blot, GAPDH was used as an internal control. Data are expressed as means ± standard deviation. *p < 0.05, **p < 0.01, ***p < 0.001.

The relative expression level of *SBSN* mRNA was evaluated in the 80 PTC tissues and paired paraneoplastic normal tissues. The qRT-PCR results showed that the relative expression level of *SBSN* mRNA was significantly higher in the PTC tissues than that in the paraneoplastic normal tissues (8.228 ± 1.452 and 5.037 ± .783, respectively; *p* < 0.05) (Figure 1A). Western blot results showed that the expression level of SBSN in the 80 PTC tissues was significantly higher than that in the paraneoplastic tissues (2.218 ± .343 and 1.563 ± .279, respectively; *p* < 0.05) (Figures 1B,C). Immunohistochemical staining showed that SBSN protein was mainly expressed in the cytoplasm (Figure 1D). The high-positive expression rate of SBSN was 12.5% (10/80) in the PTC tissues and 0% (0/80) in the adjacent normal tissues (*p* = .001). In addition, the expression of SBSN in the 9 patients with PDTC and 18 patients with ATC was detected by immunohistochemical staining. The results showed that the high-positive expression rate of SBSN in ATC (88.9%, 16/18) was significantly higher (*p* < 0.05) than those in PDTC (44.4%, 4/9) and PTC (12.5%, 10/80). Compared with that in PTC, the high-positive expression rate of SBSN was significantly higher (*p* < 0.05) in the PDTC group (Table 1). The immunohistochemical staining scores are shown in Figure 1E.

**TABLE 1:** Expression of *SBSN* in different types of thyroid tissue.

Group	*n*	Low		High		High positive rate
		(−)	(+)	(++)	(+++)	(%)
ATC	18	2	0	5	11	88.89^a^ ^,^ ^b^
PDTC	9	1	4	1	3	44.44^c^
PTC	80	12	58	8	2	12.5^d^
Normal	80	61	19	0	0	0

a ATC, vs. PDTC (*p = 0.023).

b ATC, vs. PTC (***p < 0.001).

c PDTC, vs. PTC (*p = 0.044).

d PTC, vs Normal (***p = 0.001).

### Relationships Between High SBSN Expression Level and Clinicopathological Characteristics of Patients With PTC

We characterized the clinicopathological characteristics, including the expression levels of SBSN, in 80 PTC patients. As shown in Table 2, the expression levels of *SBSN* mRNA were significantly different between the groups with and without lymph node metastasis (9.84 ± 1.91 and 4.87 ± 1.93, respectively; *p* < 0.05) (Figure 1F). The SBSN protein expression level (2.71 ± .49 vs. 1.19 ± .20) and high-positive expression rate (18.52 vs. 0%) were significantly higher (*p* < .05) in the lymph node metastasis group than in the non-metastasis group, respectively. Other clinicopathological characteristics, such as age, sex, multifocality of PTC, extra-envelope infiltration, tumor size, and clinicopathological stage and grade, were not associated with the relative expression level of SBSN (*p* > .05; Table 2). The data suggest that SBSN can influence the malignancy of PTC, thus promoting lymph node metastasis. In addition, we analyzed the relationship between *SBSN* expression and prognosis in 502 PTC patients with complete clinical information in conjunction with the TCGA database and found that *SBSN* expression had no effect on the survival time of patients (Supplementary Figure S1A), which may be attributed to the good prognosis of PTC and the low number of deaths during follow-up (16/502).

**TABLE 2:** Relationship between *SBSN* and the clinical pathological characteristics in papillary thyroid carcinoma.

Clinical features	*n*	SBSN expression [cases (%)]^a^		*p*	*SBSN* mRNA	*p*	SBSN protein^b^	*p*
		Low positive	High positive					
Gender								
Male	16	13 (81.25)	3 (18.75)	0.673	15.81 ± 5.57	0.104	1.99 ± 0.72	0.338
Female	64	57 (89.06)	7 (10.94)		6.62 ± 1.25		2.27 ± 0.39	
Age(y)								
≥55	23	20 (86.96)	3 (13.04)	0.926	8.59 ± 3.26	0.889	2.86 ± 0.91	0.301
<55	57	50 (87.72)	7 (12.28)		8.11 ± 1.63		2.02 ± 0.35	
Tumor size								
≤2	58	53 (91.38)	5 (8.62)	0.150	7.82 ± 1.75	0.949	1.86 ± 0.36	0.054
>2, ≤4	20	15 (75)	5 (25)		9.69 ± 2.84		2.61 ± 0.76	
>4	2	2 (100)	0 (0)		2.63 ± 0.10		6.71 ± 2.80	
Multifocality								
Yes	43	41 (95.35)	2 (4.65)	0.051	7.56 ± 1.90	0.758	2.22 ± 0.47	0.679
No	37	29 (78.38)	8 (21.62)		8.93 ± 2.23		2.21 ± 0.51	
Bilateral								
No	65	58 (89.23)	7 (10.77)	0.588	7.87 ± 1.70	0.418	2.30 ± 0.42	0.632
Yes	15	12 (80)	3 (20)		9.37 ± 2.82		1.95 ± 0.54	
Extrathyroid invasion								
Yes	8	6 (75)	2 (25)	0.573	8.96 ± 4.29	0.489	3.92 ± 1.88	0.521
No	72	64 (88.89)	8 (11.11)		8.13 ± 1.55		2.00 ± 0.30	
Lymph node metastasis								
Yes	54	44 (81.48)	10 (18.52)	0.047	9.84 ± 1.91	0.010	2.71 ± 0.49	0.042
No	26	26 (100)	0 (0)		4.87 ± 1.93		1.19 ± 0.20	
ACJJ stage								
Ⅰ-Ⅱ	74	65 (87.84)	9 (12.16)	0.564	8.24 ± 1.50	0.811	2.25 ± 0.36	0.771
Ⅲ-Ⅳ	6	5 (83.33)	1 (16.67)		7.99 ± 4.69		1.50 ± 0.43	
Tumor grade								
G1	26	23 (88.46)	3 (13.04)	0.857	10.26 ± 3.22	0.486	1.98 ± 0.49	0.731
G2	54	47 (87.04)	7 (12.96)		7.72 ± 1.63		2.28 ± 0.41	

a Immunohistochemical staining.

b Western blot.

### Biological Interaction Networks of SBSN

**FIGURE 2:**
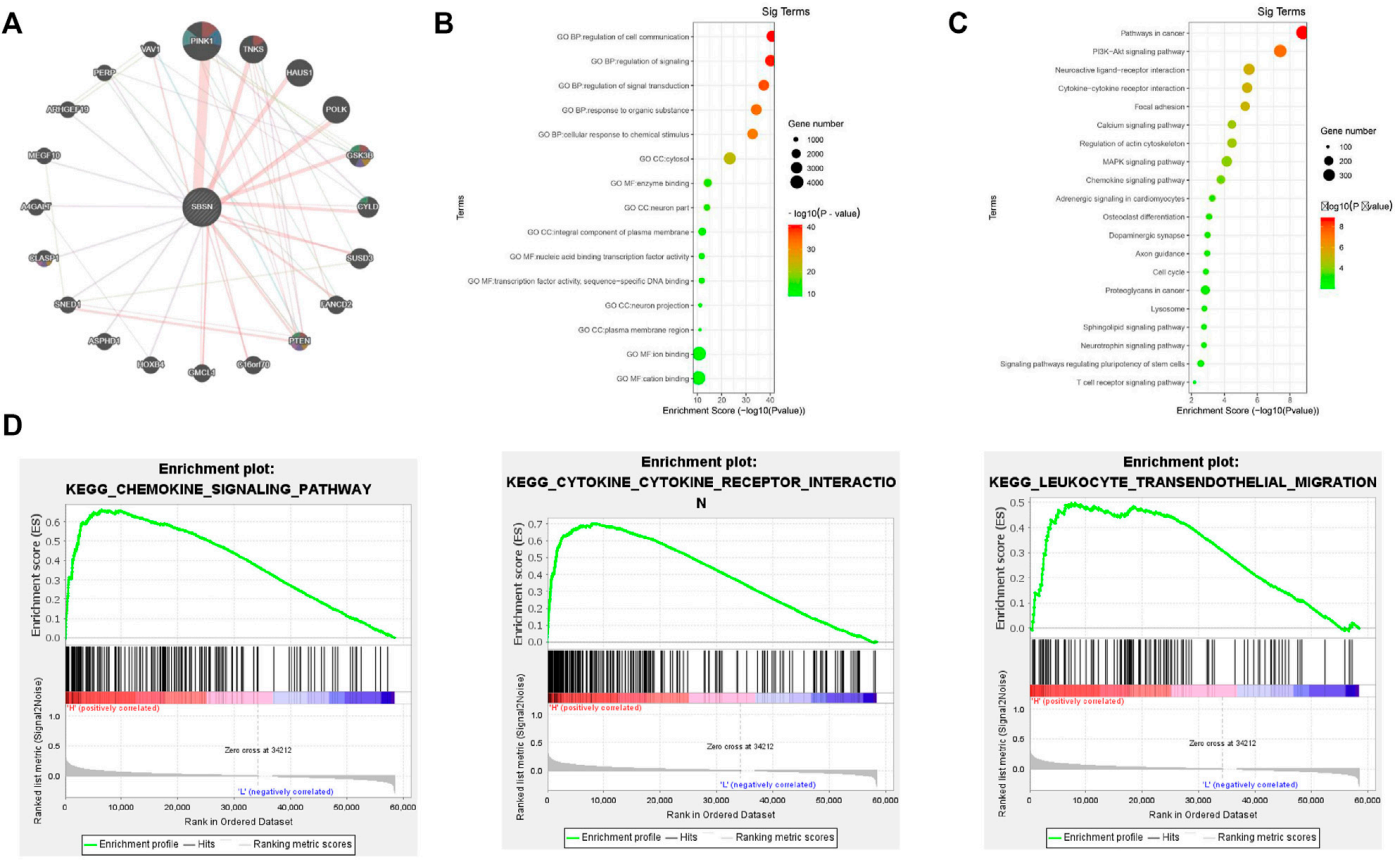
Gene interactions and enrichment analysis of *SBSN*. **(A)** Gene interaction network of the top 20 genes associated with *SBSN* in GeneMANIA. **(B)** Gene ontology enrichment analysis by genes co-expressed with *SBSN* (top five of each type). **(C)** Kyoto Encyclopedia of Genes and Genomes enrichment analysis by genes co-expressed with *SBSN*. **(D)** Gene set enrichment analysis showing *SBSN* in the published thyroid cancer dataset (The *Cancer* Genome Atlas database).

We identified the top 20 genes associated with *SBSN* through the GeneMANIA website, which showed SBSN as the central node surrounded by 20 other nodes (Figure 2A). The five most relevant genes included PTEN-inducible putative kinase 1 (*PINK1*), tankyrase (*TNKS*), HAUS augmin-like complex subunit 1 (*HAUS1*), DNA polymerase kappa (*POLK*), and glycogen synthase kinase 3β (*GSK3B*), all of which physically interacted with *SBSN*. Further functional analysis showed that these genes were associated with protein hydrolysis, amino acid modification, cell adhesion, and regulation of apoptosis. In addition, using the DAVID tool and the “ggplot2” R package, we performed functional and pathway enrichment analyses of genes in the cBioPortal database that were co-expressed with SBSN to view the biological functions and pathways associated with *SBSN*. Gene Ontology enrichment analysis revealed that *SBSN* co-expressed genes were associated with a variety of processes, including regulation of signaling, regulation of cellular signal transduction and communication, and cellular responses to chemical stimuli. They were also associated with cellular components, including the neuronal fraction and plasma membrane, and molecular functions, such as enzyme binding, activation of nucleic acid binding transcription factors, and binding of some ions (Figure 2B). Meanwhile, the enrichment of Kyoto Encyclopedia of Genes and Genomes pathways of *SBSN*-associated genes suggested that *SBSN* was associated with the PI3K/AKT, mitogen-activated protein kinase (MAPK), and other pathways commonly found in tumors. Interestingly, the cytokine receptor interaction pathway, chemokine signaling pathway, and T-cell receptor signaling pathway, which are associated with tumor immunity, were also enriched in *SBSN* co-expressed genes (Figure 2C). Similar results were obtained by ssGSEA of the published thyroid dataset (TCGA), wherein samples with high *SBSN* expression levels were associated with levels of cytokines and chemokines and more pronounced leukocyte migration (Figure 2D).

### Correlation of SBSN Expression Levels With Immune and Stromal Cells in the TME

**FIGURE 3:**
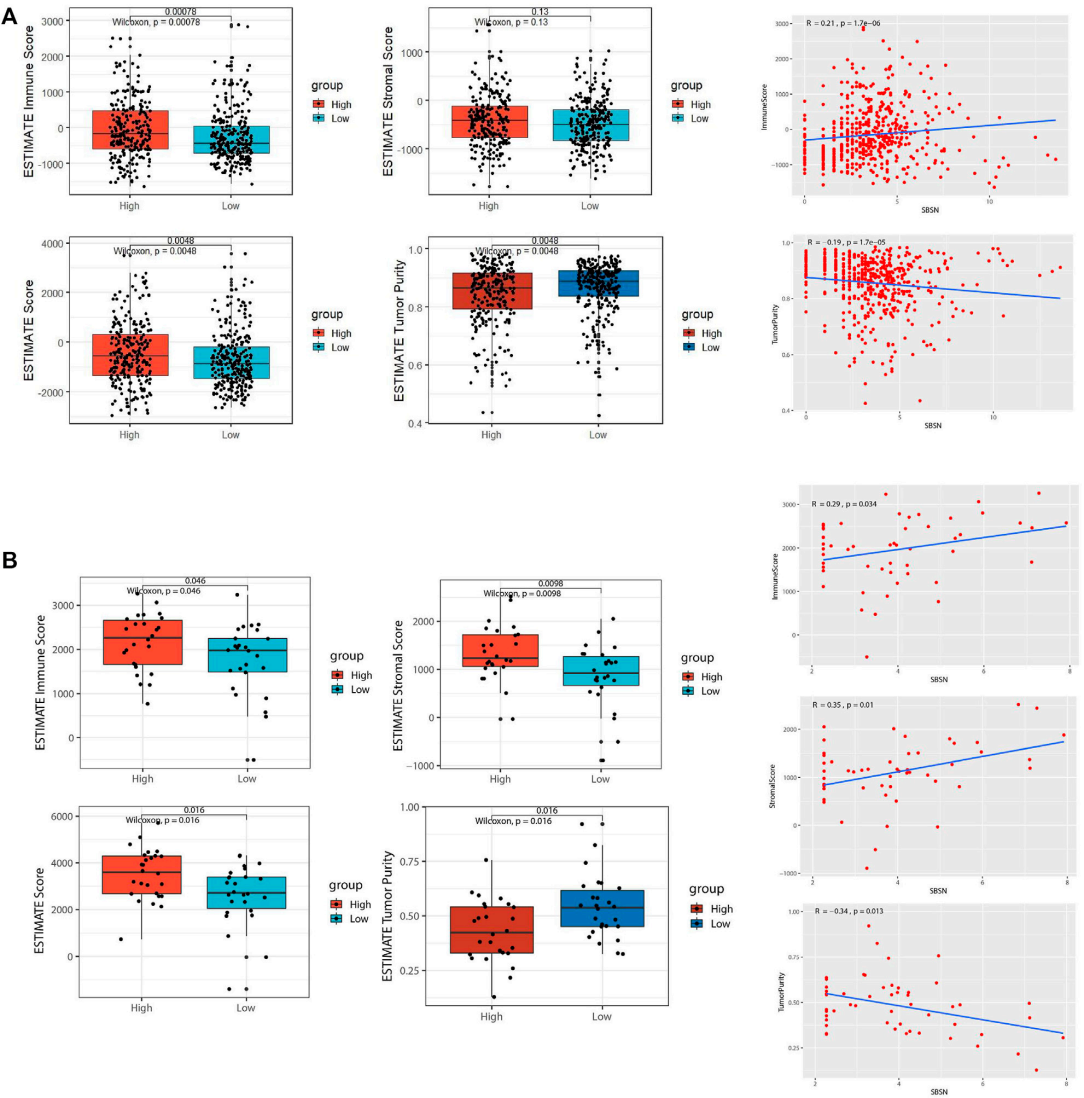
Comparison of tumor microenvironment (TME) scores and *SBSN* expression profiles in follicular epithelial cell-derived thyroid cancer. **(A)** Comparison of the immune score, stromal score, estimate score, and tumor purity in *SBSN* ^high^ and *SBSN* ^low^ papillary thyroid carcinoma tissue groups as calculated by the ESTIMATE algorithm. **(B)** Comparison of the immune score, stromal score, estimate score, and tumor purity in *SBSN* ^high^ and *SBSN* ^low^ anaplastic thyroid carcinoma tissue groups as calculated by the ESTIMATE algorithm. Differences between *SBSN* ^high^ and *SBSN* ^low^ groups were compared using two-sided Wilcoxon rank-sum test.

To assess the distribution of immune and stromal cells in PTC, PDTC, and ATC samples, we analyzed the 502 primary PTC samples from the TCGA dataset; 52 ATC samples from GSE29265, GSE33630, GSE76039, and GSE65144; and 22 PDTC samples from GSE53157 and GSE76039. Based on the median values of *SBSN* expression levels in the TCGA PTC cohort, PDTC joint cohort, and ATC joint cohort, the samples were divided into *SBSN* ^high^ and *SBSN* ^low^ groups. Based on the ESTIMATE algorithm, we found that, in the PTC samples, the immune score was higher in the *SBSN* ^high^ group than in the *SBSN* ^low^ group (*p* < 0.01), while *SBSN* expression level was negatively correlated with tumor purity (*p* < 0.01). There was no significant association between the stromal scores and SBSN expression levels (Figure 3A). In the ATC samples, the immune and stromal scores were higher in the *SBSN* ^high^ group than in the *SBSN* ^low^ group (*p* < 0.05), and *SBSN* expression level was negatively correlated with the extent of tumor purity (*p* < 0.05; Figure 3B). In the PDTC samples, no significant associations were found between *SBSN* expression levels and the immune scores, stromal scores, or degree of tumor purity (Supplementary Figure S1B), which may have been due to the small sample size. In conclusion, these findings suggest that *SBSN* expression may impact immune cells in the PTC TME and immune and stromal cells in the ATC TME.

### Relationship Between SBSN Expression Level and Immune Cell Infiltration

**FIGURE 4:**
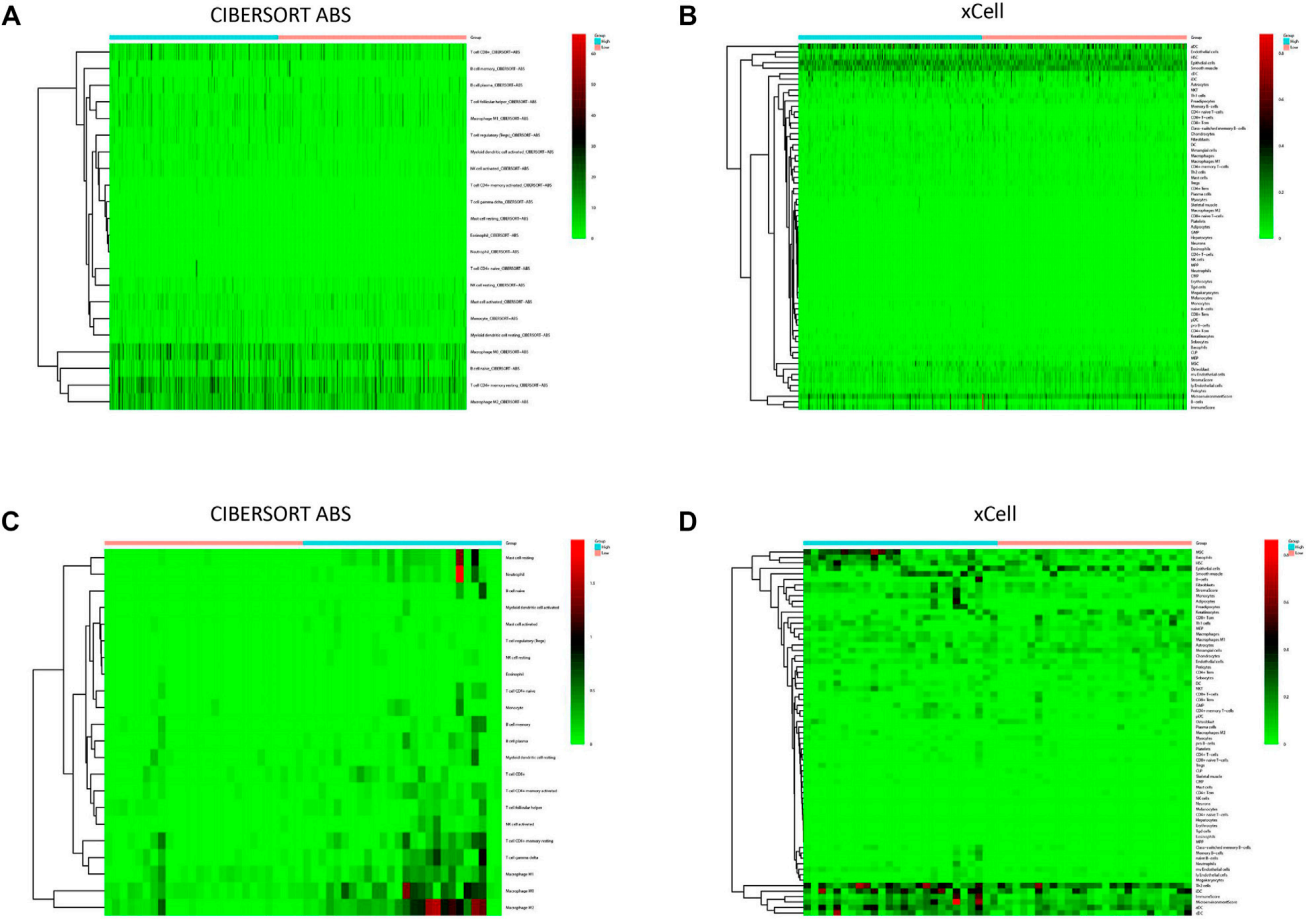
Immune cell infiltration in patients with papillary thyroid carcinoma (PTC) and anaplastic thyroid carcinoma (ATC). **(A)** Heat map of tumor-infiltrating immune cell (TIIC) fractions, quantified by CIBERSORT-ABS, in PTC. **(B)** Heat map of TIIC fractions, quantified by xCell, in PTC. **(C)** Heat map of TIIC fractions, quantified by CIBERSORT-ABS, in ATC. **(D)** Heat map of TIIC fractions, quantified by xCell, in ATC.

**FIGURE 5:**
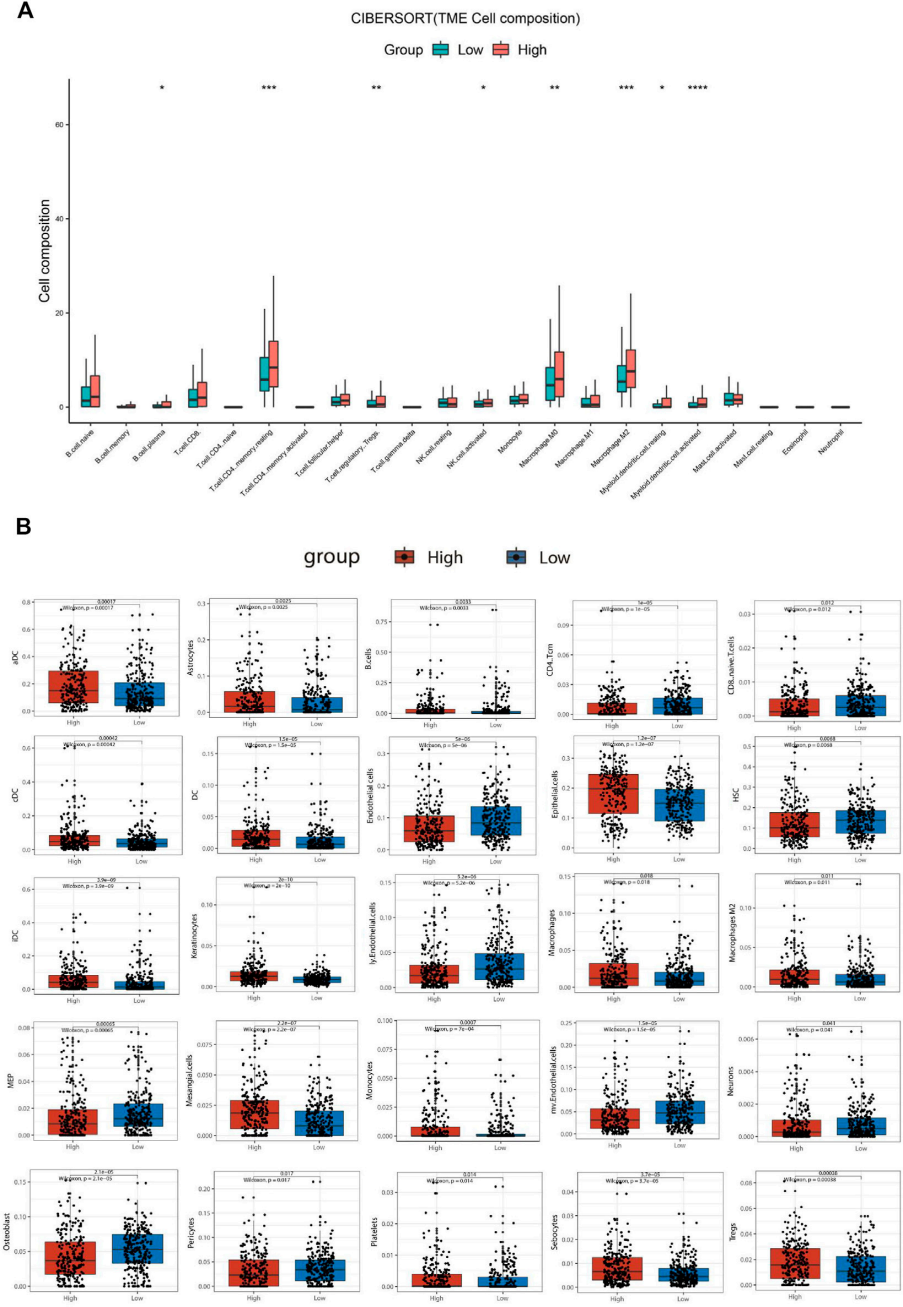
Comparison of different non-cancer cell proportions, quantified by CIBERSORT-ABS algorithm and xCell algorithm, in *SBSN* ^high^ and *SBSN* ^low^ papillary thyroid carcinoma groups. **(A)** Comparison of different immune cell proportions, quantified by the CIBERSORT-ABS algorithm, in the *SBSN* ^high^ and *SBSN* ^low^ groups. **(B)** Comparison of different non-cancer cell proportions, quantified by the xCell algorithm, in the *SBSN* ^high^ and *SBSN* ^low^ groups. Differences between *SBSN* ^high^ and *SBSN* ^low^ groups were compared using the two-sided Wilcoxon rank-sum test. *p < .05, **p < .01, ***p < .001, ****p < .0001.

To explore the correlation between *SBSN* expression and the immune microenvironment, we inferred the abundances of TIICs using CIBERSORT-ABS and xCell. The association between *SBSN* expression and TIICs in PTC and ATC is shown in Figure 4. In the PTC samples, the proportions of TIIC types were significantly different between patients in the *SBSN* ^high^ and *SBSN* ^low^ groups (Figures 4A,B). Similarly, the proportions of TIICs in the ATC samples were significantly different between the *SBSN* ^high^ and *SBSN* ^low^ groups (Figures 4C,D). In addition, we sought to determine whether the tumor immune microenvironment was different in patients with different *SBSN* expression levels. For the PTC samples, the results of the deconvolution algorithm CIBERSORT-ABS showed that the proportions of effector B cells (*p* < 0.05), resting CD4^+^ memory T cells (*p* < 0.001), Tregs (*p* < 0.01), activated natural killer (NK) cells (*p* < 0.05), M0 macrophages (*p* < 0.01), M2 macrophages (*p* < .001), resting dendritic cells (DCs) (*p* < .05), and activated DCs (*p* < .0001) were significantly elevated in the *SBSN* ^high^ group (Figure 5A). The results of the xCell algorithm showed that 25 out of 64 noncancerous cell types were correlated and 39 cell types were not correlated with *SBSN* expression (Supplementary Figure S2). Among the former, 15 types had higher proportions in the *SBSN* ^high^ group, and 10 types had higher proportions in the *SBSN* ^low^ group. Numbers of activated DCs (*p* < .01), B cells (*p* < .01), conventional DCs (*p* < .01), DCs (*p* < .01), immature DCs (iDCs) (*p* < .01), macrophages (*p* < .05), M2 macrophages (*p* < .05), monocytes (*p* < .01), and Tregs (*p* < .01) were all significantly elevated in the *SBSN* ^high^ group, while those of central memory CD4^+^ T cells (*p* < .01) and CD8^+^ naïve T cells (*p* < .05) were significantly elevated in the *SBSN* ^low^ group. In addition, some other cell types, such as keratin-forming cells (*p* < .01), epithelial cells (*p* < .01), platelets (*p* < .05), astrocytes (*p* < .01), mesangial cells (*p* < .01), and sebocytes (*p* < .01), were more prevalent in the *SBSN* ^high^ group; meanwhile, endothelial cells (*p* < .01), ly endothelial cells (*p* < .01), hematopoietic stem cells (HSC) (*p* < .01), megakaryocyte–erythroid progenitor (MEP) (*p* < .01), multinucleated variant endothelial cells (*p* < .01), neurons (*p* < .05), osteoblasts (*p* < .01), and pericytes (*p* < .05) were more predominant in the *SBSN* ^low^ group (Figure 5B).

**FIGURE 6:**
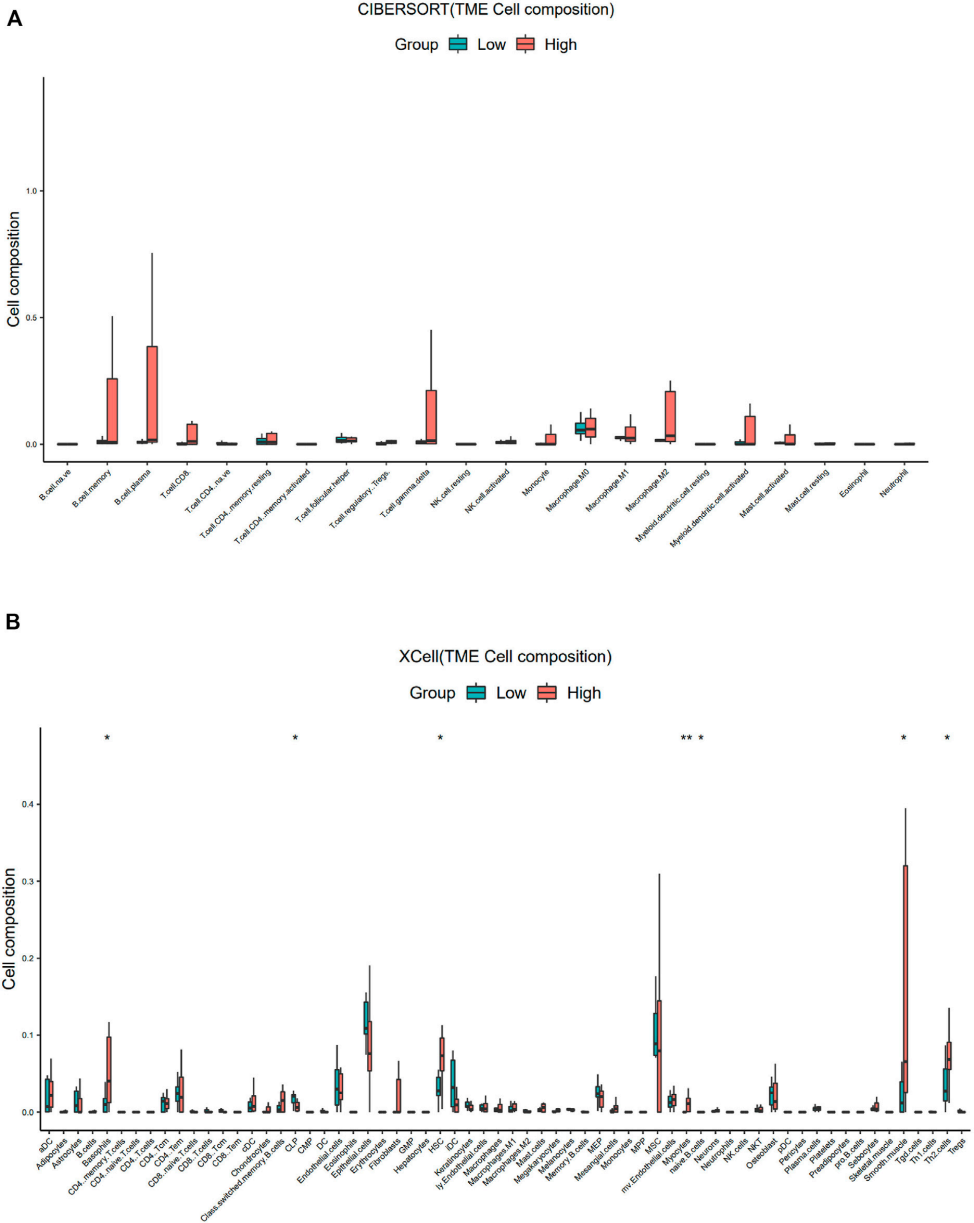
Comparison of different non-cancer cell proportions, quantified by CIBERSORT-ABS algorithm and xCell algorithm, in *SBSN* ^high^ and *SBSN* ^low^ poorly differentiated thyroid carcinoma groups. **(A)** Comparison of different immune cell proportions, quantified by the CIBERSORT-ABS algorithm, in the *SBSN* ^high^ and *SBSN* ^low^ groups. **(B)** Comparison of different non-cancer cell proportions, quantified by the xCell algorithm, in the *SBSN* ^high^ and *SBSN* ^low^ groups. Differences between *SBSN* ^high^ and *SBSN* ^low^ groups were compared using the two-sided Wilcoxon rank-sum test. *p < .05, **p < .01, ***p < .001, ****p < .0001.

Similar to the results of ESTIMATE analysis, the CIBERSORT-ABS results showed no correlation between the immune cell content in PDTC and *SBSN* expression (Figure 6A), while xCell analysis yielded higher proportions of only basophils (*p* < .05), hematopoietic stem cells (*p* < .05), myocytes (*p* < .01), smooth muscle cells (*p* < .05), and T helper (Th) cell type 2 (Th2) cells (*p* < .05) in the *SBSN* ^high^ group. The *SBSN* ^low^ group had a higher proportion of common lymphoid progenitors (*p* < .05) as well as naïve B cells (*p* < .05) (Figure 6B).

**FIGURE 7:**
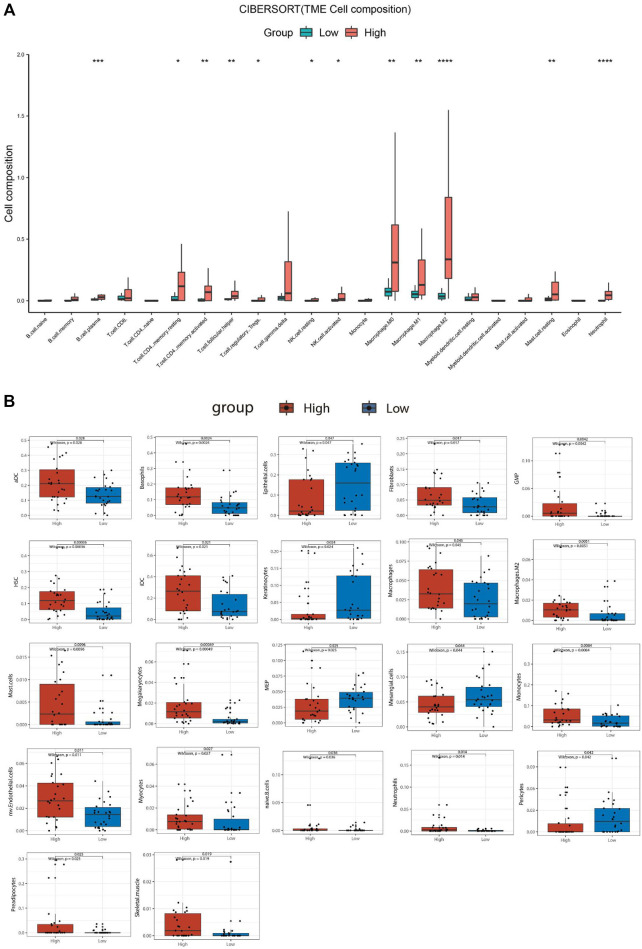
Comparison of different non-cancer cell proportions, quantified by CIBERSORT-ABS algorithm and xCell algorithm, in *SBSN* ^high^ and *SBSN* ^low^ anaplastic thyroid carcinoma groups. **(A)** Comparison of different immune cell proportions, quantified by the CIBERSORT-ABS algorithm, in the *SBSN* ^high^ and *SBSN* ^low^ groups. **(B)** Comparison of different non-cancer cell proportions, quantified by the xCell algorithm, in the *SBSN* ^high^ and *SBSN* ^low^ groups. Differences between *SBSN* ^high^ and *SBSN* ^low^ groups were compared using the two-sided Wilcoxon rank-sum test. *p < .05, **p < .01, ***p < .001, ****p < .0001.

For the ATC samples, the CIBERSORT-ABS results showed that effector B cells (*p* < .001), resting CD4^+^ memory T cells (*p* < .05), activated CD4^+^ memory T cells (*p* < .01), follicular helper T cells (*p* < .01), Tregs (*p* < .05), resting NK cells (*p* < .05), activated NK cells (*p* < .05), macrophages (*p* < .01), M1 macrophages (*p* < .01), M2 macrophages (*p* < .0001), resting mast cells (*p* < .01), and neutrophils (*p* < .0001) were significantly more numerous in the *SBSN* ^high^ group (Figure 7A). The xCell algorithm showed that 22 out of the 64 noncancerous cell types were correlated and 42 cell types were not correlated with *SBSN* expression (Supplementary Figure S3). Among the 22 cell types, 17 had higher proportions in the *SBSN* ^high^ group and five in the *SBSN* ^low^ group. Proportions of activated DCs (*p* < .05), basophils (*p* < .01), iDCs (*p* < .05), macrophages (*p* < .05), M2 macrophages (*p* < .01), mast cells (*p* < .01), monocytes (*p* < .01), naïve B cells (*p* < .05), and neutrophils (*p* < .05) were significantly elevated in the *SBSN* ^high^ group. Some other cell types, such as fibroblasts (*p* < .05), granulocyte–macrophage lineage progenitors (*p* < .01), hematopoietic stem cells (*p* < .01), megakaryocytes (*p* < .01), multinucleated variant endothelial cells (*p* < .05), myocytes (*p* < .05), preadipocytes (*p* < .05), and skeletal muscle cells (*p* < .05) were also more common in the *SBSN* ^high^ group. Meanwhile, epithelial cells (*p* < .05), keratin-forming cells (*p* < .05), megakaryocyte–erythroid progenitor cells (*p* < .05), mesangial cells (*p* < .05), and pericytes (*p* < .05) were more highly represented in the *SBSN* ^low^ group (Figure 7B). Taken together, these results suggest that SBSN can regulate different types of tumor-associated cells in PTC as well as in the ATC TME.

### Correlation of SBSN Expression Levels in PTC and ATC Tumor Tissues With the Densities of CD163^+^ M2 Macrophages and Foxp3^+^ Tregs

**FIGURE 8:**
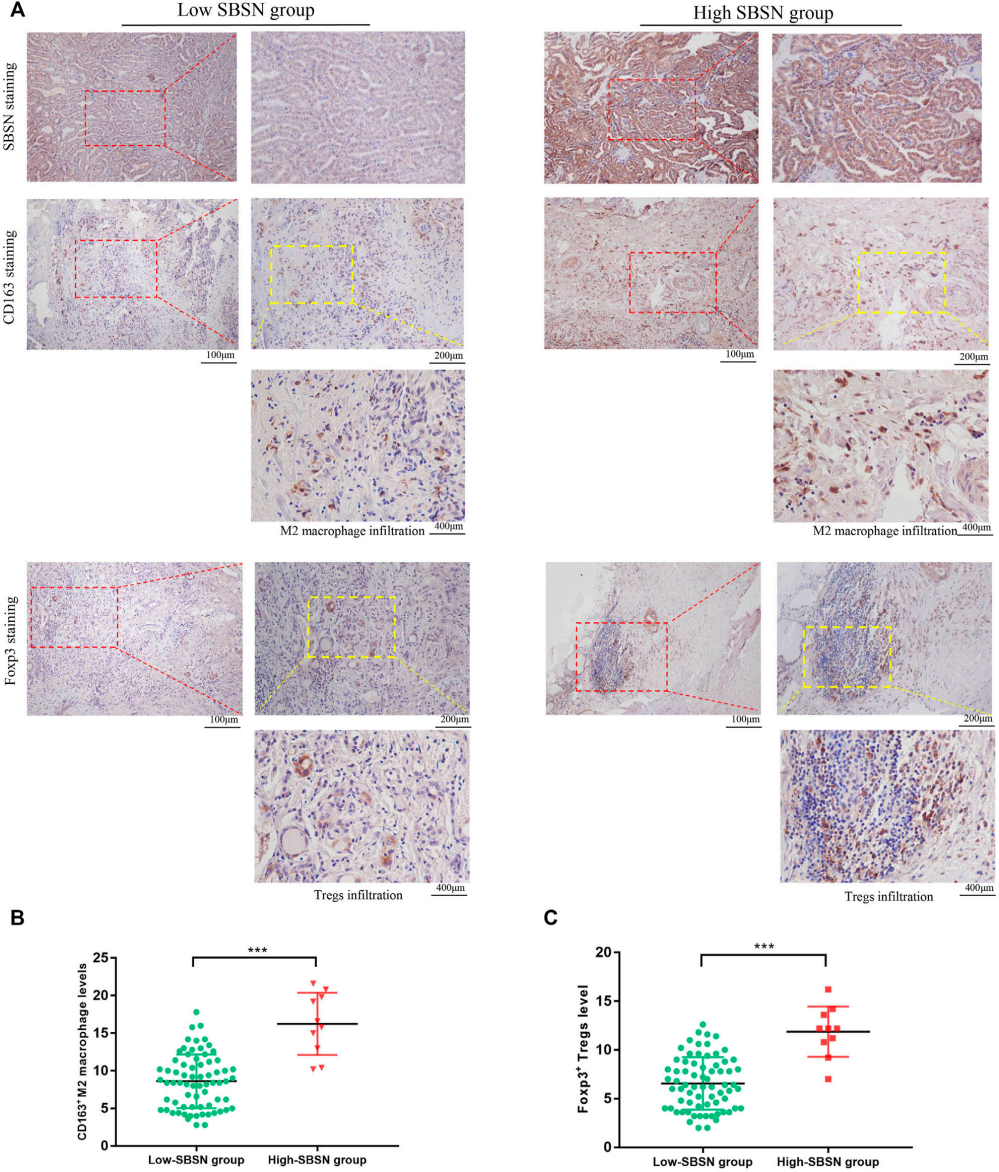
The correlation of SBSN expression with CD163^+^ M2 macrophages and Foxp3^+^ T regulatory cells (Tregs) in patients with papillary thyroid carcinoma (PTC) was analyzed by immunohistochemical staining. **(A)** Immunohistochemical staining of SBSN, CD163, and Foxp3 detected M2 macrophage and Treg infiltration in SBSN low-positive expression and SBSN high-positive expression groups. **(B)** Scatter-plot of CD163^+^ M2 macrophage density in the low- and high-positive SBSN expression PTC groups. **(C)** Scatter-plot of Foxp3^+^ Treg density in the low- and high-positive SBSN expression PTC groups. Results are presented as means ± standard deviation. ***p < .001.

**FIGURE 9:**
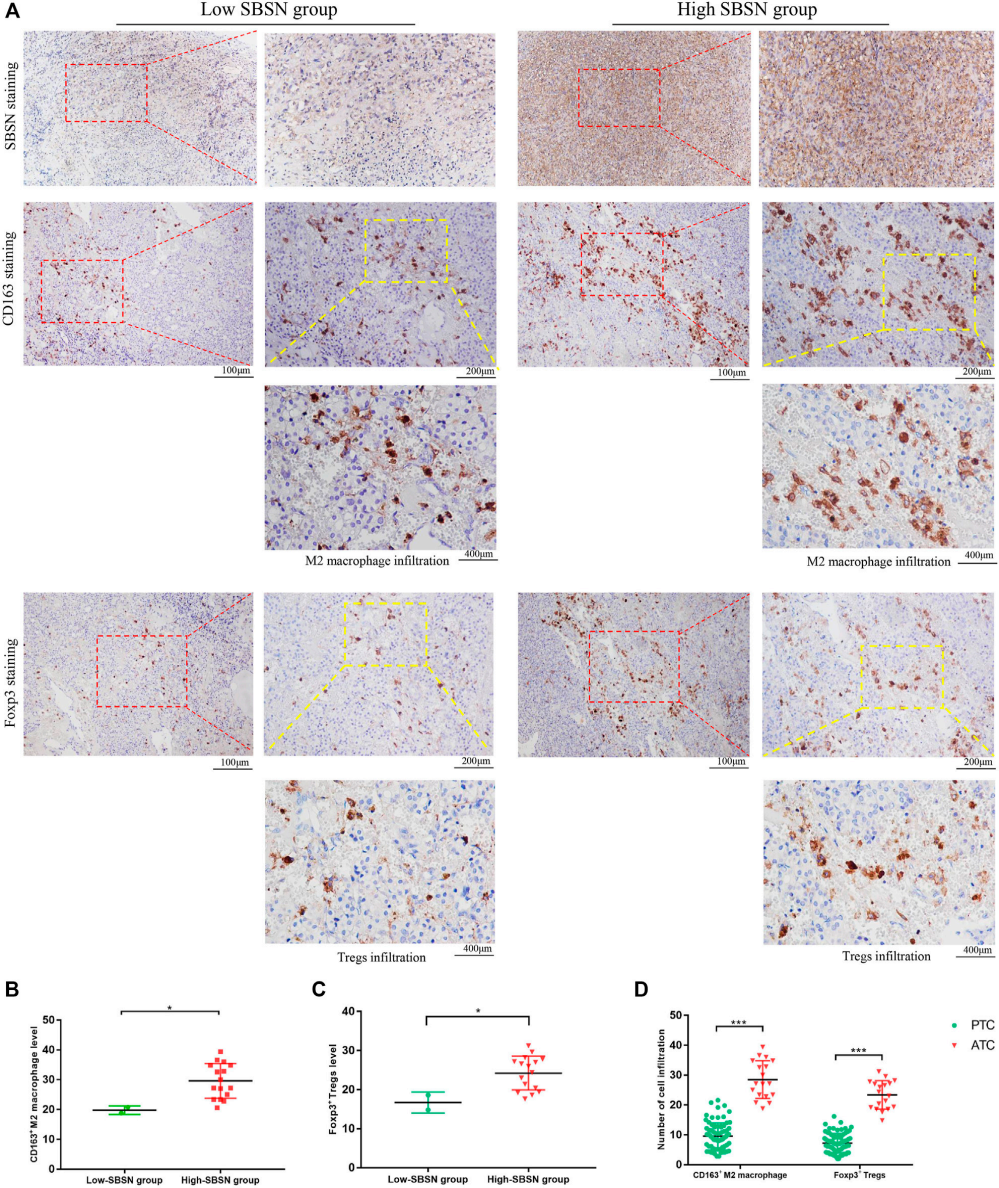
The correlation of SBSN expression with CD163^+^ M2 macrophages and Foxp3^+^ T regulatory cells (Tregs) in patients with anaplastic thyroid carcinoma (ATC) was analyzed by immunohistochemical staining. **(A)** Immunohistochemical staining of SBSN, CD163, and Foxp3 detected M2 macrophage and Treg infiltration in SBSN low-positive expression and SBSN high-positive expression groups. **(B)** Scatter-plot of CD163^+^ M2 macrophage density in the low- and high-positive SBSN expression ATC groups. **(C)** Scatter-plot of Foxp3^+^ Treg density in the low- and high-positive SBSN expression ATC groups. **(D)** Scatter-plots of CD163^+^ M2 macrophage and Foxp3^+^ Treg densities in papillary thyroid carcinoma (PTC) and ATC. Results are presented as means ± standard deviation. *p < .05, ***p < .001.

To confirm the results obtained by comprehensive bioinformatics analysis, we performed immunohistochemical staining of tumor tissues from 80 patients with PTC and 18 patients with ATC for the M2 macrophage marker CD163 and the Treg marker Foxp3. CD163+M2 macrophages and Foxp3+Tregs were mainly distributed in the interstitium of tumor tissues and lymphocyte aggregates in PTC (Figure 8A) and mainly scattered around cancer cells in ATC (Figure 9A). The results showed that, in PTC tissues, the infiltration levels of CD163+M2 macrophages (16.24 ± 4.315) and Foxp3+Tregs (11.88 ± 2.581) were higher in the SBSN high-positive expression group than in the SBSN low-positive expression group (8.606 ± 3.566 and 6.566 ± 2.69, respectively; *p* < .01) (Figures 8B,C). In ATC tissues, the infiltration levels of CD163+M2 macrophages (29.6 ± 5.791) and Foxp3+Tregs (24.23 ± 4.318) were higher in the SBSN high-positive expression group than in the SBSN low-positive expression group (19.8 ± 1.414 and 16.7 ± 2.687, respectively; *p* < .05) (Figures 9B,C). In addition, the results of staining of CD163+M2 macrophages and Foxp3+Tregs in 80 cases of PTC and 18 of ATC showed that the total infiltration levels of CD163+M2 macrophages (28.51 ± 6.305) and Foxp3+Tregs (23.39 ± 4.775) were higher in ATC than in PTC (9.56 ± 4.417 and 7.23 ± 3.194, respectively; *p* < .01) (Figure 9D).

### Association of *SBSN* Expression With the Cancer Immunity Cycle

**FIGURE 10:**
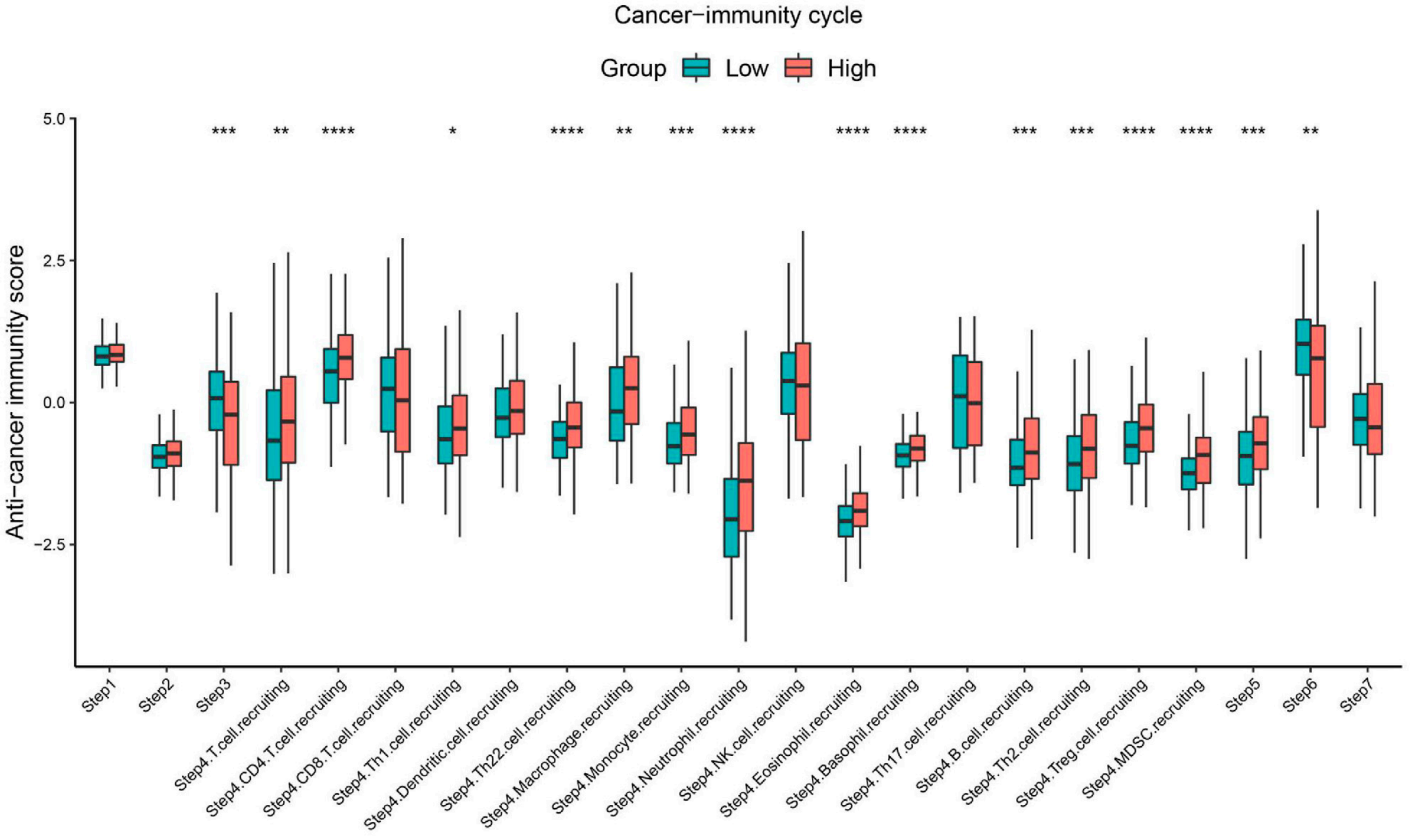
Comparison between the *SBSN* ^high^ and *SBSN* ^low^ groups in the scores for the cancer immunity cycle in PTC. The thick line represents the median value within each group, and differences between the *SBSN* ^high^ and *SBSN* ^low^ groups were compared using the two-sided Wilcoxon rank-sum test. *p < .05, **p < .01, ***p < .001, ****p < .0001.

The cancer immunity cycle involves a series of steps that enable the anti-cancer immune response to kill cancer cells effectively: tumor cell release of antigen (step I), tumor antigen presentation (step II), T cell activation (step III), T cell migration to tumor tissue (step IV), tumor tissue T cell infiltration (step V), T cell recognition of tumor cells (step VI), and clearance of tumor cells (step VII) (Chen and Mellman, 2013). The TIP pipeline was used to estimate the activity score of SBSN in the seven-step cancer immunity cycle in PTC samples. The results showed that the *SBSN* ^high^ group had higher scores (*p* < .05) than those of the *SBSN* ^low^ group in the processes of T cell recruitment and infiltration of recruited T cells into tumor tissue. These included CD4^+^ T cells, Th1 cells, Th22 cells, macrophages, monocytes, neutrophils, eosinophils, basophils, B cells, Th2 cells, Tregs, and bone marrow-derived suppressor cells (MDSCs). However, the *SBSN* ^high^ group had lower anti-cancer immune scores (*p* < .05) during T-cell activation and T-cell recognition of tumor cells (Figure 10). Overall, these results suggest a possible role of *SBSN* in the cancer immunity cycle of PTC.

## Discussion

This study aimed to predict the role of SBSN in follicular epithelial cell-derived thyroid cancer using an extensive bioinformatics data mining approach to explore the relationship between SBSN expression levels and the extent of infiltration by different immune cells. To our knowledge, the present study is the first to highlight the relationship between SBSN expression and immune infiltration in thyroid cancer, providing new insights into the role of SBSN in cancer-associated immune regulation and its application as a cancer biomarker.

SBSN was originally identified in epithelial tissues and is thought to be involved in the process of epidermal differentiation under physiological conditions (Park et al., 2002). In recent years, accumulating evidence has indicated that SBSN plays an important role in the development of a variety of tumors. Hypomethylation of the *SBSN* promoter leads to elevated *SBSN* mRNA levels in NSCLC, and aberrant SBSN expression promotes the proliferation of lung squamous cell lines (Glazer et al., 2009). Similarly, in salivary ACC, the CpG island is hypomethylated in *SBSN*, and knockdown of *SBSN* inhibits the proliferation and invasive ability of ACC cell lines (Shao et al., 2012). In ESCC, high SBSN expression level shortens patient survival, and overexpression of SBSN enhances the proliferation and invasive ability of esophageal cancer cells, while *SBSN* knockdown does the opposite (Zhu et al., 2016). In malignant brain tumors, SBSN upregulation is associated with poor prognosis for patients with glioblastoma multiforme (Formolo et al., 2011). Tumor development cannot be separated from angiogenesis, and SBSN is involved in the migration of tumor endothelial cells and angiogenesis through AKT activation (Alam et al., 2014; Takahashi et al., 2020). All of these previous findings suggest that *SBSN* is involved as an oncogene in tumor progression.

In our study, the possible role of SBSN in follicular epithelial cell-derived thyroid cancer and the involved regulatory mechanisms were explored. SBSN was highly expressed in PTC at the protein and mRNA levels. SBSN expression was significantly associated with cervical-lymph-node metastasis in PTC (*p* < .05). These results suggest that SBSN expression may be an indicator to assess the aggressiveness of PTC. Importantly, SBSN expression levels increased with decreasing extent of differentiation and increasing rates of malignancy in follicular epithelial cell-derived thyroid cancer; high-positive expression levels were most pronounced in ATC. This finding indicates that SBSN may be involved in regulating the malignancy of tumors and that its expression level may be related to the degree of tumor malignancy, accurately reflecting the progression of tumor malignancy.

We used two types of enrichment analysis to further elucidate the potential mechanisms of *SBSN*’s involvement in follicular-derived thyroid cancer. *SBSN* and its co-expressed genes were associated with a variety of biological processes, cellular components, and molecular functions, especially signaling, regulation, and responses to stimuli. *SBSN* was also found to be associated with several cancer signaling pathways, such as the PI3K/AKT and MAPK pathways; this result is consistent with those of previous studies (Alam et al., 2014; Takahashi et al., 2020). Interestingly, SBSN expression was also significantly enriched in several cancer immunity-related pathways, such as the chemokine pathway, the cytokine and its receptor interaction pathway, and the T-cell receptor signaling pathway, which are usually involved in tumorigenic processes (Liu T. et al., 2012; Li and Rudensky, 2016). These results suggest that *SBSN* may play complex roles in multiple biological processes.

A deeper analysis of the complexity within the TME may help identify patient populations with the potential to respond to current immune checkpoint therapies and may contribute to the identification of new adjuvant therapeutic targets (Lin et al., 2019). Previously, several studies have reported that elevated immune scores are associated with poor prognosis in patients with different cancers, such as renal cell carcinoma and osteosarcoma (Xu et al., 2019; Zhang et al., 2020), while stromal cells are also thought to play an important role in tumor growth, disease progression, and drug resistance (Denton et al., 2018). Therefore, we explored the relationship between TME scores and SBSN expression using the ESTIMATE algorithm. Our study is the first to demonstrate that, in PTC, SBSN expression level was positively correlated with immune scores and negatively correlated with tumor purity, while no significant association was observed between stromal scores and SBSN expression levels. In ATC, SBSN expression level was significantly positively correlated with immune and stromal scores and negatively correlated with tumor purity. These results suggest that SBSN may contribute to increasing numbers of immune and stromal cells in the TME, which diminishes tumor purity. Indeed, the elevated immune and stromal scores in the TME may indicate either protection or a poor prognosis for the host depending on the type of immune cells that infiltrate the tumor and the specific roles they play in tumor development (Cunha et al., 2014).

The types and relative proportions of TIICs in the TME may correlate with the clinical prognosis of patients (Orhan et al., 2020). Based on the functional enrichment analysis of SBSN and the correlation of its expression with immune and stromal cells in the TME, we further explored the relevance of SBSN expression to immune cell infiltration. Large amounts of RNA-seq data have enabled algorithms that employ the deconvolution principle or gene markers to map the TME of samples. Since there is no gold standard for inferring immune infiltration from RNA-seq data, we selected two algorithms, based on the deconvolution principle and the gene marker principle, to infer the composition of TIICs and confirmed their correlation by immunohistochemical staining. The proportion of M2 macrophages was higher in the *SBSN* ^high^ group in both the PTC and ATC samples. The proportion of M2 macrophages in the *SBSN* ^high^ group was much higher than that in the *SBSN* ^low^ group and higher than that of M1 macrophages in the *SBSN* ^high^ group in the ATC samples, suggesting that macrophages may be more polarized to the M2 phenotype in ATC. In most tumors, M2 macrophages are present as immunosuppressive cells, which can release growth factors to promote tumor development (Ho et al., 2016). More importantly, M2 macrophages tend to promote neoangiogenesis as well as stromal activation and remodeling (Afik et al., 2016), thus positively influencing cancer progression and negatively affecting patient prognosis (Tiainen et al., 2015). PTC tissues have been shown to have high levels of immunity, especially due to M2 macrophages, and compared with early PTC, advanced PTC exhibits a higher degree of immune infiltration and a higher proportion of M2 macrophages, which accelerate tumor cell migration (Zhang et al., 2021), producing a pro-cancer effect and exacerbating immune escape (Xie et al., 2020). Therefore, a high expression level of SBSN in patients with PTC and ATC may accelerate tumor progression by stimulating the polarization of M2 macrophages.

Furthermore, both CIBERSORT-ABS and xCell results showed that SBSN expression was positively correlated with the percentages of multiple DCs, including iDCs, and Tregs in PTC samples. Similarly, in ATC tissues, the percentage of iDCs was positively correlated with SBSN expression. The numbers of Tregs and DCs have been shown to increase in PTC tissues (Yu et al., 2013). Usually, tumor-infiltrating DCs exhibit an immature phenotype, resulting in altered antigen presentation (Tran Janco et al., 2015). iDCs do not effectively induce T- and NK-cell-mediated immune responses and can even suppress immune responses by producing suppressive cytokines, such as IL-10 and TGF-β (Scouten and Francis, 2006). In addition, Tregs, as well as TAMs, alter the normal replication of endothelial cells by creating a hypoxic environment in the tumor tissue and can achieve immunosuppressive and escape effects by inhibiting the antigen presentation by DCs and the activation of CD8^+^ T cells in the tumor (Facciabene et al., 2017; Jang et al., 2017). The proportion of Tregs in PTC also correlates with lymph node metastasis and extrathyroidal expansion (French et al., 2010), which is consistent with our results. These findings suggest that patients with PTC and ATC with high SBSN expression levels may benefit from targeting SBSN to reduce the proportions of Tregs and iDCs.

In addition, SBSN expression levels in ATC samples were positively correlated with the proportions of mast cells and neutrophils, unlike those in PTC. The role of neutrophils in cancer is still controversial, but in recent years, thyroid cancer cells have been shown to be able to recruit neutrophils by releasing CXCL8/IL-8, improve their own survival by releasing granulocyte colony-stimulating factor, enhancing the pro-inflammatory response of neutrophils, and upregulate the expression of pro-oncogenic factors (Galdiero et al., 2018). Regarding mast cells in tumors, a previous study showed that the presence of mast cells in ATC correlates with tumor aggressiveness and that mast cells induce epithelial-mesenchymal transition in thyroid cancer cell lines, mainly by activating CXCL8 in the AKT/SLUG pathway (Visciano et al., 2015). These results suggest that SBSN may be involved in the regulation of multiple infiltrating immune cells in the process of tumor development and contribute to tumor progression.

Interestingly, as suggested by the ESTIMATE algorithm, our exploration in PDTC yielded different results from those for ATC and PTC. CIBERSORT-ABS and xCell results showed a low level of immune cell infiltration in PDTC, and most immune cells and stromal cells did not correlate with *SBSN* expression. This is partly due to the small sample size of PDTC compared with those of PTC and ATC. However, we found similar results in the study by Giannini et al. (2019) who found that PDTC showed poor or absent immune cell infiltration compared with that in ATC and PTC, that the degree of immune cell infiltration in PDTC was even lower than that in normal thyroid tissue, and that, in most cases, PDTC appeared as non–T-cell-inflamed “cold” tumors.

Furthermore, our results showed that, in the xCell analysis of PTC, compared with the *SBSN* ^high^ group, the *SBSN* ^low^ group had a higher proportion of central memory CD4^+^ T cells as well as CD8^+^ naïve T cells. Central memory CD4^+^ T cells were reported to have stronger anti-tumor capacity compared with that of effector memory CD4^+^ T cells (Klebanoff et al., 2005). They have a strong self-renewal and replication ability and not only survive for a long time *in vivo* but also can be efficiently expanded *in vitro* to ensure the number of T cells returned for infusion, which can play a long-term anti-tumor role (Zhou et al., 2005; Berger et al., 2008). Naïve CD8^+^ T cells will differentiate into many effector cells when encountering antigens such as tumor cells, and these effector cells migrate to the corresponding sites to produce antitumor effects (Brummelman et al., 2018). The decrease in these cellular components coupled with an increase in *SBSN* expression indicates that the *in vivo* anti-tumor capacity is weakened and that tumor cells have a greater chance to develop immune escape ability.

Our results also demonstrated that some blood cells showed differential expression in the *SBSN* ^high^ and *SBSN* ^low^ groups. HSCs were able to promote tumor growth and progression in the solid TME (Hassan and Seno, 2020). However, the correlation between *SBSN* expression and HSCs in PTC and ATC produced opposite results, which may be attributed to the difference in the degree of malignancy of the two subtypes. Compared with PTC, ATC usually exhibits strong invasive and metastatic abilities, which are dependent on the supply of hematopoietic cells, reflecting the pro-carcinogenic role of *SBSN* in ATC. We also observed that the content of MEPs in ATC and PTC decreased with the expression of *SBSN*. Usually, in solid tumors or leukemia, MEPs can be transformed into erythroblast-like cells, erythrocytes, or megakaryocytes, thus promoting tumor progression (Wickrema and Crispino, 2007; Han et al., 2018). This implies that elevated *SBSN* expression may lead to increased conversion of MEPs into the aforementioned cells and may promote tumor progression.

In addition, studies on the dedifferentiation of thyroid cancer have gained interest in recent years. A previous study showed that immune scores are significantly negatively correlated with thyroid cancer differentiation scores (Na and Choi, 2018) and that the least differentiated ATC usually has higher stromal and immune scores than those for highly differentiated PTC (Cunha et al., 2021), which suggests that the immune microenvironment may be involved in the process of thyroid cancer dedifferentiation. In our study, the xCell results showed that SBSN expression in ATC was positively correlated with fibroblast content in tumors, while no such relationship was found in PTC. Wen et al. (2021) found that cancer-associated fibroblast (CAF) content was significantly higher in ATC than in PTC or normal thyroid tissue. The content of CAFs was positively correlated with dedifferentiation, aggressiveness, and poor prognosis of thyroid cancer, which suggests that SBSN may promote dedifferentiation processes and contribute to poor outcomes of thyroid cancer by regulating the content of CAFs. In addition, M2 macrophages can activate the Wnt/β-catenin pathway by secreting Wnt1 and Wnt3a, participating in the dedifferentiation, migration, and proliferation of invasive thyroid cancer cells (Lv et al., 2021). Our study indicated that SBSN expression level was positively correlated with the numbers of M2 macrophages in both PTC and ATC; however, compared with that in PTC, the proportion of M2 macrophages in the *SBSN* ^high^ ATC group was significantly higher than that in the *SBSN* ^low^ group. These findings suggest that SBSN may promote the dedifferentiation and aggressiveness of thyroid cancer cells by regulating the content of M2 macrophages, especially in ATC.

Regarding the cancer immunity cycle, patients with PTC with high SBSN expression levels scored higher in T-cell migration to tumor tissue (step IV) and tumor tissue T-cell infiltration (step V). However, according to the results of the cancer immunity cycle, most of the recruited and infiltrated immune cells, such as TAMs, neutrophils, and Tregs, have immunosuppressive effects on the tumor. There are also some helper T cells, such as Th2 cells, whose secreted cytokines can mediate the polarization of macrophages into the M2 phenotype (Shapouri-Moghaddam et al., 2018), and the increase in Th22 cell number has been reported to be associated with the progression of gastric cancer (Liu Z. et al., 2012). In addition, the recruitment of monocytes and MDSCs is positively correlated with SBSN expression in the cancer immunity cycle. Monocytes have been shown to be direct precursors of HSC-derived macrophages, and upon recruitment to tumor tissue, they can differentiate into TAMs and support tumorigenesis, local progression, and distant metastasis (Richards et al., 2013). MDSCs are rare in healthy subjects, but their numbers are elevated in patients with cancer, in whom they show a potent immunosuppressive potential and are associated with a poor prognosis (Marvel and Gabrilovich, 2015). Furthermore, patients with PTC with high SBSN expression levels showed lower scores in processes such as T cell activation and T cell recognition of tumor cells, which might be due to higher levels of iDCs and Tregs in their tissues, as DCs with an immature phenotype are usually unable to fully activate T cells (Yu et al., 2013) and Tregs also suppress the activation of CD8^+^ T cells (Jang et al., 2017). In addition, iDCs have abnormally altered antigen-presenting functions (Tran Janco et al., 2015), and Tregs in tumors also suppress antigen-presenting functions of DCs, leading to impaired T-cell recognition (Jang et al., 2017). These results imply that SBSN may suppress T-cell activation and inhibit T-cell recognition by affecting the levels of DCs and Tregs and may contribute to the immune escape of tumor cells.

There are several limitations to the current study. First, more thyroid cancer tissue samples are needed to validate the relationships between SBSN expression and immune and stromal cells in the TME and further explore the correlation between SBSN expression and immune cell infiltration. Second, there is no canonical method to analyze infiltrating immune cells in the TME, and we used two different methods that are based on different principles. Thus, additional studies are needed to elucidate the mechanism of SBSN’s effects on immune cell infiltration in thyroid cancer. Third, RNA-seq-based algorithms may not be sufficiently accurate; overcoming this limitation requires *in vivo* models to explore the underlying biological mechanisms of SBSN’s effects and its interaction with tumor immunity in thyroid cancer.

## Conclusion

In conclusion, our study showed that SBSN is highly expressed in follicular epithelial cell-derived thyroid cancer. The expression level of SBSN increases with decreasing extent of cancer cell differentiation and is associated with lymph node metastasis in patients with PTC, which can be used as a potential biomarker for follicular epithelial cell-derived thyroid cancer. In addition, SBSN can influence the cancer immunity cycle and promote thyroid cancer dedifferentiation by regulating the level of immune cell infiltration in the TME. This suggests that *SBSN* may be a therapeutic target whose inhibition may promote anti-tumor immune response. Thus, a comprehensive understanding of the relationship between SBSN expression and immune infiltration may provide new insights into the immunotherapy of thyroid cancer.

## Data Availability

The original contributions presented in the study are included in the article/Supplementary Material, further inquiries can be directed to the corresponding author.

## Supplementary Material

Supplementary Figure S1
**(A)** Survival curves of 502 patients with papillary thyroid carcinoma in the Cancer Genome Atlas (TCGA) database. **(B)** Comparison between *SBSN* ^high^ and *SBSN* ^low^ poorly differentiated thyroid carcinoma groups in the immune score, stromal score, estimate score, and tumor purity, calculated by ESTIMATE algorithm.

Supplementary Figure S2 Non-cancerous cells that do not correlate with SBSN expression as derived by the xCell algorithm in papillary thyroid carcinoma.

Supplementary Figure S3 Non-cancerous cells that do not correlate with *SBSN* expression as derived by the xCell algorithm in anaplastic thyroid carcinoma.

## Abbreviations

ACC, adenoid cystic carcinoma; ATC, anaplastic thyroid carcinoma; CAF, cancer-associated fibroblast; DCs, dendritic cells; ESCC, esophageal squamous cell carcinoma; GSEA, gene set enrichment analysis; HSCs, hematopoietic stem cells; iDCs, immature dendritic cells; MDSCs, marrow-derived suppressor cells; MEP, megakaryocyte–erythroid progenitor; NSCLC, non-small cell lung cancer; PDTC, poorly differentiated thyroid carcinoma; PTC, papillary thyroid carcinomas; qRT-PCR, quantitative reverse-transcription polymerase chain reaction; SBSN, suprabasin; ssGSEA, single-sample gene set enrichment analysis; TAMs, tumor-associated macrophages; TCGA, the *cancer* genome atlas; Th2, T helper cell type 2; TIICs, tumor-infiltrating immune cells; TIP, tumor immunophenotype; TME, tumor microenvironment; Tregs, regulatory T cells.

## References

[B1] Abdullah M. I. Junit S. M. Ng K. L. Jayapalan J. J. Karikalan B. Hashim O. H. (2019). Papillary Thyroid Cancer: Genetic Alterations and Molecular Biomarker Investigations. Int. J. Med. Sci. 16 (3), 450–460. 10.7150/ijms.29935 30911279 PMC6428975

[B2] Afik R. Zigmond E. Vugman M. Klepfish M. Shimshoni E. Pasmanik-Chor M. (2016). Tumor Macrophages Are Pivotal Constructors of Tumor Collagenous Matrix. J. Exp. Med. 213 (11), 2315–2331. 10.1084/jem.20151193 27697834 PMC5068227

[B3] Alam M. T. Nagao‐Kitamoto H. Ohga N. Akiyama K. Maishi N. Kawamoto T. (2014). Suprabasin as a Novel Tumor Endothelial Cell Marker. Cancer Sci. 105 (12), 1533–1540. 10.1111/cas.12549 25283635 PMC4317965

[B4] Aoshima M. Phadungsaksawasdi P. Nakazawa S. Iwasaki M. Sakabe J.-i. Umayahara T. (2019). Decreased Expression of Suprabasin Induces Aberrant Differentiation and Apoptosis of Epidermal Keratinocytes: Possible Role for Atopic Dermatitis. J. Dermatol. Sci. 95 (3), 107–112. 10.1016/j.jdermsci.2019.07.009 31399284

[B5] Aran D. Hu Z. Butte A. J. (2017). xCell: Digitally Portraying the Tissue Cellular Heterogeneity Landscape. Genome Biol. 18 (1), 220. 10.1186/s13059-017-1349-1 29141660 PMC5688663

[B6] Baxevanis C. N. Sofopoulos M. Fortis S. P. Perez S. A. (2019). The Role of Immune Infiltrates as Prognostic Biomarkers in Patients with Breast Cancer. Cancer Immunol. Immunother. 68 (10), 1671–1680. 10.1007/s00262-019-02327-7 30905043 PMC11028310

[B7] Berger C. Jensen M. C. Lansdorp P. M. Gough M. Elliott C. Riddell S. R. (2008). Adoptive Transfer of Effector CD8+ T Cells Derived from central Memory Cells Establishes Persistent T Cell Memory in Primates. J. Clin. Invest. 118 (1), 294–305. 10.1172/JCI32103 18060041 PMC2104476

[B8] Brummelman J. Pilipow K. Lugli E. (2018). The Single-Cell Phenotypic Identity of Human CD8+ and CD4+ T Cells. Int. Rev. Cel Mol Biol. 341, 63–124. 10.1016/bs.ircmb.2018.05.007 30262035

[B9] Cancer Genome Atlas Research Network (2014). Integrated Genomic Characterization of Papillary Thyroid Carcinoma. Cell 159 (3), 676–690. 10.1016/j.cell.2014.09.050 25417114 PMC4243044

[B10] Capdevila J. Mayor R. Mancuso F. M. Iglesias C. Caratù G. Matos I. (2018). Early Evolutionary Divergence between Papillary and Anaplastic Thyroid Cancers. Ann. Oncol. 29 (6), 1454–1460. 10.1093/annonc/mdy123 29648575

[B11] Chen D. S. Mellman I. (2013). Oncology Meets Immunology: The Cancer-Immunity Cycle. Immunity 39 (1), 1–10. 10.1016/j.immuni.2013.07.012 23890059

[B12] Cunha L. L. Domingues G. A. B. Morari E. C. Soares F. A. Vassallo J. Ward L. S. (2021). The Immune Landscape of the Microenvironment of Thyroid Cancer Is Closely Related to Differentiation Status. Cancer Cel Int. 21 (1), 387. 10.1186/s12935-021-02084-7 PMC8293508 34284788

[B13] Cunha L. L. Marcello M. A. Ward L. S. (2014). The Role of the Inflammatory Microenvironment in Thyroid Carcinogenesis. Endocr. Relat. Cancer 21 (3), R85–R103. 10.1530/ERC-13-0431 24302667

[B14] Denton A. E. Roberts E. W. Fearon D. T. (2018). Stromal Cells in the Tumor Microenvironment. Adv. Exp. Med. Biol. 1060, 99–114. 10.1007/978-3-319-78127-3_6 30155624

[B15] Dralle H. Machens A. Basa J. Fatourechi V. Franceschi S. Hay I. D. (2015). Follicular Cell-Derived Thyroid Cancer. Nat. Rev. Dis. Primers 1, 15077. 10.1038/nrdp.2015.77 27188261

[B16] Facciabene A. De Sanctis F. Pierini S. Reis E. S. Balint K. Facciponte J. (2017). Local Endothelial Complement Activation Reverses Endothelial Quiescence, Enabling T-Cell Homing, and Tumor Control during T-Cell Immunotherapy. Oncoimmunology 6 (9), e1326442. 10.1080/2162402X.2017.1326442 28932632 PMC5599081

[B17] Fang W. Ye L. Shen L. Cai J. Huang F. Wei Q. (2014). Tumor-associated Macrophages Promote the Metastatic Potential of Thyroid Papillary Cancer by Releasing CXCL8. Carcinogenesis 35 (8), 1780–1787. 10.1093/carcin/bgu060 24608042

[B18] Formolo C. A. Williams R. Gordish-Dressman H. MacDonald T. J. Lee N. H. Hathout Y. (2011). Secretome Signature of Invasive Glioblastoma Multiforme. J. Proteome Res. 10 (7), 3149–3159. 10.1021/pr200210w 21574646 PMC3136381

[B19] French J. D. Kotnis G. R. Said S. Raeburn C. D. McIntyre R. C. Klopper J. P. (2012). Programmed Death-1+ T Cells and Regulatory T Cells Are Enriched in Tumor-Involved Lymph Nodes and Associated with Aggressive Features in Papillary Thyroid Cancer. J. Clin. Endocrinol. Metab. 97 (6), E934–E943. 10.1210/jc.2011-3428 22466343 PMC3387418

[B20] French J. D. Weber Z. J. Fretwell D. L. Said S. Klopper J. P. Haugen B. R. (2010). Tumor-associated Lymphocytes and Increased Foxp3+ Regulatory T Cell Frequency Correlate with More Aggressive Papillary Thyroid Cancer. J. Clin. Endocrinol. Metab. 95 (5), 2325–2333. 10.1210/jc.2009-2564 20207826 PMC2869546

[B21] Galdiero M. R. Varricchi G. Loffredo S. Bellevicine C. Lansione T. Ferrara A. L. (2018). Potential Involvement of Neutrophils in Human Thyroid Cancer. PLoS ONE 13, e0199740. 10.1371/journal.pone.0199740 29953504 PMC6023126

[B22] Giannini R. Moretti S. Ugolini C. Macerola E. Menicali E. Nucci N. (2019). Immune Profiling of Thyroid Carcinomas Suggests the Existence of Two Major Phenotypes: An ATC-like and a PDTC-like. J. Clin. Endocrinol. Metab. 104 (8), 3557–3575. 10.1210/jc.2018-01167 30882858

[B23] Glazer C. A. Smith I. M. Ochs M. F. Begum S. Westra W. Chang S. S. (2009). Integrative Discovery of Epigenetically Derepressed Cancer Testis Antigens in NSCLC. PLoS One 4 (12), e8189. 10.1371/journal.pone.0008189 19997593 PMC2781168

[B24] Gospodarowicz M. Mackillop W. O'Sullivan B. Sobin L. Henson D. Hutter R. V. (2001). Prognostic Factors in Clinical Decision Making: the Future. Cancer 91 (8 Suppl. l), 1688–1695. 10.1002/1097-0142(20010415)91:8+<1688:aid-cncr1184>3.0.co;2-7 11309769

[B25] Han Y. Liu Q. Hou J. Gu Y. Zhang Y. Chen Z. (2018). Tumor-Induced Generation of Splenic Erythroblast-like Ter-Cells Promotes Tumor Progression. Cell 173 (3), 634–648. 10.1016/j.cell.2018.02.061 29606356

[B26] Hassan G. Seno M. (2020). Blood and Cancer: Cancer Stem Cells as Origin of Hematopoietic Cells in Solid Tumor Microenvironments. Cells 9 (5), 1293. 10.3390/cells9051293 PMC7290570 32455995

[B27] Ho V. W. Hofs E. Elisia I. Lam V. Hsu B. E. Lai J. (2016). All Trans Retinoic Acid, Transforming Growth Factor β and Prostaglandin E2 in Mouse Plasma Synergize with Basophil-Secreted Interleukin-4 to M2 Polarize Murine Macrophages. PLoS One 11 (12), e0168072. 10.1371/journal.pone.0168072 27977740 PMC5158015

[B28] Ichinose K. Ohyama K. Furukawa K. Higuchi O. Mukaino A. Satoh K. (2018). Novel Anti-suprabasin Antibodies May Contribute to the Pathogenesis of Neuropsychiatric Systemic Lupus Erythematosus. Clin. Immunol. 193, 123–130. 10.1016/j.clim.2017.11.006 29162406

[B29] Jang J.-E. Hajdu C. H. Liot C. Miller G. Dustin M. L. Bar-Sagi D. (2017). Crosstalk between Regulatory T Cells and Tumor-Associated Dendritic Cells Negates Anti-tumor Immunity in Pancreatic Cancer. Cel Rep. 20 (3), 558–571. 10.1016/j.celrep.2017.06.062 PMC5649374 28723561

[B30] Jung K. Y. Cho S. W. Kim Y. A. Kim D. Oh B.-C. Park D. J. (2015). Cancers with Higher Density of Tumor-Associated Macrophages Were Associated with Poor Survival Rates. J. Pathol. Transl Med. 49 (4), 318–324. 10.4132/jptm.2015.06.01 26081823 PMC4508569

[B31] Klebanoff C. A. Gattinoni L. Torabi-Parizi P. Kerstann K. Cardones A. R. Finkelstein S. E. (2005). Central Memory Self/tumor-Reactive CD8+ T Cells Confer superior Antitumor Immunity Compared with Effector Memory T Cells. Proc. Natl. Acad. Sci. 102 (27), 9571–9576. 10.1073/pnas.0503726102 15980149 PMC1172264

[B32] La Vecchia C. Malvezzi M. Bosetti C. Garavello W. Bertuccio P. Levi F. (2015). Thyroid Cancer Mortality and Incidence: a Global Overview. Int. J. Cancer 136 (9), 2187–2195. 10.1002/ijc.29251 25284703

[B33] Landa I. Ibrahimpasic T. Boucai L. Sinha R. Knauf J. A. Shah R. H. (2016). Genomic and Transcriptomic Hallmarks of Poorly Differentiated and Anaplastic Thyroid Cancers. J. Clin. Invest. 126 (3), 1052–1066. 10.1172/JCI85271 26878173 PMC4767360

[B34] Li M. O. Rudensky A. Y. (2016). T Cell Receptor Signalling in the Control of Regulatory T Cell Differentiation and Function. Nat. Rev. Immunol. 16 (4), 220–233. 10.1038/nri.2016.26 27026074 PMC4968889

[B35] Lin P. Guo Y.-n. Shi L. Li X.-j. Yang H. He Y. (2019). Development of a Prognostic index Based on an Immunogenomic Landscape Analysis of Papillary Thyroid Cancer. Aging 11 (2), 480–500. 10.18632/aging.101754 30661062 PMC6366981

[B36] Liu T. Peng L. Yu P. Zhao Y. Shi Y. Mao X. (2012a). Increased Circulating Th22 and Th17 Cells Are Associated with Tumor Progression and Patient Survival in Human Gastric Cancer. J. Clin. Immunol. 32 (6), 1332–1339. 10.1007/s10875-012-9718-8 22760549

[B37] Liu Z. Sun D.-X. Teng X.-Y. Xu W.-X. Meng X.-P. Wang B.-S. (2012b). Expression of Stromal Cell-Derived Factor 1 and CXCR7 in Papillary Thyroid Carcinoma. Endocr. Pathol. 23 (4), 247–253. 10.1007/s12022-012-9223-x 23070788

[B38] Lote H. Cafferkey C. Chau I. (2015). PD-1 and PD-L1 Blockade in Gastrointestinal Malignancies. Cancer Treat. Rev. 41 (10), 893–903. 10.1016/j.ctrv.2015.09.004 26412280

[B39] Lv J. Feng Z. P. Chen F. K. Liu C. Jia L. Liu P. J. (2021). M2‐like Tumor‐associated Macrophages‐secreted Wnt1 and Wnt3a Promotes Dedifferentiation and Metastasis via Activating β‐catenin Pathway in Thyroid Cancer. Mol. Carcinogenesis 60 (1), 25–37. 10.1002/mc.23268 33283877

[B40] Marvel D. Gabrilovich D. I. (2015). Myeloid-derived Suppressor Cells in the Tumor Microenvironment: Expect the Unexpected. J. Clin. Invest. 125 (9), 3356–3364. 10.1172/JCI80005 26168215 PMC4588239

[B41] Mazzaferri E. L. Jhiang S. M. (1994). Long-term Impact of Initial Surgical and Medical Therapy on Papillary and Follicular Thyroid Cancer. Am. J. Med. 97 (5), 418–428. 10.1016/0002-9343(94)90321-2 7977430

[B42] Melillo R. M. Guarino V. Avilla E. Galdiero M. R. Liotti F. Prevete N. (2010). Mast Cells Have a Protumorigenic Role in Human Thyroid Cancer. Oncogene 29 (47), 6203–6215. 10.1038/onc.2010.348 20729915

[B43] Molinaro E. Romei C. Biagini A. Sabini E. Agate L. Mazzeo S. (2017). Anaplastic Thyroid Carcinoma: from Clinicopathology to Genetics and Advanced Therapies. Nat. Rev. Endocrinol. 13 (11), 644–660. 10.1038/nrendo.2017.76 28707679

[B44] Motzer R. J. Escudier B. McDermott D. F. George S. Hammers H. J. Srinivas S. (2015). Nivolumab versus Everolimus in Advanced Renal-Cell Carcinoma. N. Engl. J. Med. 373 (19), 1803–1813. 10.1056/NEJMoa1510665 26406148 PMC5719487

[B45] Na K. J. Choi H. (2018). Immune Landscape of Papillary Thyroid Cancer and Immunotherapeutic Implications. Endocr. Relat. Cancer 25 (5), 523–531. 10.1530/ERC-17-0532 29507047

[B46] Newman A. M. Liu C. L. Green M. R. Gentles A. J. Feng W. Xu Y. (2015). Robust Enumeration of Cell Subsets from Tissue Expression Profiles. Nat. Methods 12 (5), 453–457. 10.1038/nmeth.3337 25822800 PMC4739640

[B47] Orhan A. Vogelsang R. P. Andersen M. B. Madsen M. T. Hölmich E. R. Raskov H. (2020). The Prognostic Value of Tumour-Infiltrating Lymphocytes in Pancreatic Cancer: a Systematic Review and Meta-Analysis. Eur. J. Cancer 132, 71–84. 10.1016/j.ejca.2020.03.013 32334338

[B48] Papp S. Asa S. L. (2015). When Thyroid Carcinoma Goes Bad: a Morphological and Molecular Analysis. Head Neck Pathol. 9 (1), 16–23. 10.1007/s12105-015-0619-z 25804379 PMC4382495

[B49] Park G. T. Lim S. E. Jang S.-I. Morasso M. I. (2002). Suprabasin, a Novel Epidermal Differentiation Marker and Potential Cornified Envelope Precursor. J. Biol. Chem. 277 (47), 45195–45202. 10.1074/jbc.m205380200 12228223 PMC1283087

[B50] Reck M. Rodríguez-Abreu D. Robinson A. G. Hui R. Csőszi T. Fülöp A. (2016). Pembrolizumab versus Chemotherapy for PD-L1-Positive Non-small-cell Lung Cancer. N. Engl. J. Med. 375 (19), 1823–1833. 10.1056/NEJMoa1606774 27718847

[B51] Richards D. M. Hettinger J. Feuerer M. (2013). Monocytes and Macrophages in Cancer: Development and Functions. Cancer Microenvironment 6 (2), 179–191. 10.1007/s12307-012-0123-x 23179263 PMC3717063

[B52] Scouten W. T. Francis G. L. (2006). Thyroid Cancer and the Immune System: a Model for Effective Immune Surveillance. Expert Rev. Endocrinol. Metab. 1 (3), 353–366. 10.1586/17446651.1.3.353 30764074

[B53] Shao C. Tan M. Bishop J. A. Liu J. Bai W. Gaykalova D. A. (2012). Suprabasin Is Hypomethylated and Associated with Metastasis in Salivary Adenoid Cystic Carcinoma. PLoS One 7 (11), e48582. 10.1371/journal.pone.0048582 23144906 PMC3492451

[B54] Shapouri‐Moghaddam A. Mohammadian S. Vazini H. Taghadosi M. Esmaeili S. A. Mardani F. (2018). Macrophage Plasticity, Polarization, and Function in Health and Disease. J. Cel Physiol. 233 (9), 6425–6440. 10.1002/jcp.26429 PMC13482560 29319160

[B55] Takahashi K. Asano N. Imatani A. Kondo Y. Saito M. Takeuchi A. (2020). Sox2 Induces Tumorigenesis and Angiogenesis of Early-Stage Esophageal Squamous Cell Carcinoma through Secretion of Suprabasin. Carcinogenesis 41 (11), 1543–1552. 10.1093/carcin/bgaa014 32055838

[B56] Tiainen S. Tumelius R. Rilla K. Hämäläinen K. Tammi M. Tammi R. (2015). High Numbers of Macrophages, Especially M2-like (CD163-Positive), Correlate with Hyaluronan Accumulation and Poor Outcome in Breast Cancer. Histopathology 66 (6), 873–883. 10.1111/his.12607 25387851

[B57] Tran Janco J. M. Lamichhane P. Karyampudi L. Knutson K. L. (2015). Tumor-infiltrating Dendritic Cells in Cancer Pathogenesis. J.I. 194 (7), 2985–2991. 10.4049/jimmunol.1403134 PMC4369768 25795789

[B58] Visciano C. Liotti F. Prevete N. Cali' G. Franco R. Collina F. (2015). Mast Cells Induce Epithelial-To-Mesenchymal Transition and Stem Cell Features in Human Thyroid Cancer Cells through an IL-8-Akt-Slug Pathway. Oncogene 34 (40), 5175–5186. 10.1038/onc.2014.441 25619830

[B59] Waniczek D. Lorenc Z. Śnietura M. Wesecki M. Kopec A. Muc-Wierzgoń M. (2017). Tumor-Associated Macrophages and Regulatory T Cells Infiltration and the Clinical Outcome in Colorectal Cancer. Arch. Immunol. Ther. Exp. 65 (5), 445–454. 10.1007/s00005-017-0463-9 PMC5602054 28343267

[B60] Wen S. Qu N. Ma B. Wang X. Luo Y. Xu W. (2021). Cancer-Associated Fibroblasts Positively Correlate with Dedifferentiation and Aggressiveness of Thyroid Cancer. Ott 14, 1205–1217. 10.2147/OTT.S294725 PMC7910116 33654411

[B61] Wickrema A. Crispino J. D. (2007). Erythroid and Megakaryocytic Transformation. Oncogene 26 (47), 6803–6815. 10.1038/sj.onc.1210763 17934487

[B62] Xiangqian Z. Chen P. Ming G. Jingtai Z. Xiukun H. Jingzhu Z. (2019). Risk Factors for Cervical Lymph Node Metastasis in Papillary Thyroid Microcarcinoma: a Study of 1,587 Patients. Cancer Biol. Med. 16 (1), 121–130. 10.20892/j.issn.2095-3941.2018.0125 31119052 PMC6528461

[B63] Xie Z. Li X. He Y. Wu S. Wang S. Sun J. (2020). Immune Cell Confrontation in the Papillary Thyroid Carcinoma Microenvironment. Front. Endocrinol. 11, 570604. 10.3389/fendo.2020.570604 PMC7642595 33193087

[B64] Xu B. Ghossein R. (2020). Poorly Differentiated Thyroid Carcinoma. Semin. Diagn. Pathol. 37 (5), 243–247. 10.1053/j.semdp.2020.03.003 32360274

[B65] Xu L. Deng C. Pang B. Zhang X. Liu W. Liao G. (2018). TIP: A Web Server for Resolving Tumor Immunophenotype Profiling. Cancer Res. 78 (23), 6575–6580. 10.1158/0008-5472.CAN-18-0689 30154154

[B66] Xu W.-H. Xu Y. Wang J. Wan F.-N. Wang H.-K. Cao D.-L. (2019). Prognostic Value and Immune Infiltration of Novel Signatures in clear Cell Renal Cell Carcinoma Microenvironment. Aging 11 (17), 6999–7020. 10.18632/aging.102233 31493764 PMC6756904

[B67] Yoshihara K. Shahmoradgoli M. Martínez E. Vegesna R. Kim H. Torres-Garcia W. (2013). Inferring Tumour Purity and Stromal and Immune Cell Admixture from Expression Data. Nat. Commun. 4, 2612. 10.1038/ncomms3612 24113773 PMC3826632

[B68] Yu H. Huang X. Liu X. Jin H. Zhang G. e. Zhang Q. (2013). Regulatory T Cells and Plasmacytoid Dendritic Cells Contribute to the Immune Escape of Papillary Thyroid Cancer Coexisting with Multinodular Non-toxic Goiter. Endocrine 44 (1), 172–181. 10.1007/s12020-012-9853-2 23264145

[B69] Zhang C. Gu X. Pan M. Yuan Q. Cheng H. (2021). Senescent Thyroid Tumor Cells Promote Their Migration by Inducing the Polarization of M2-like Macrophages. Clin. Transl Oncol. 23 (6), 1253–1261. 10.1007/s12094-020-02516-2 33389662

[B70] Zhang C. Zheng J.-H. Lin Z.-H. Lv H.-Y. Ye Z.-M. Chen Y.-P. (2020). Profiles of Immune Cell Infiltration and Immune-Related Genes in the Tumor Microenvironment of Osteosarcoma. Aging 12 (4), 3486–3501. 10.18632/aging.102824 32039832 PMC7066877

[B71] Zhang H. Liu H. Shen Z. Lin C. Wang X. Qin J. (2018). Tumor-infiltrating Neutrophils Is Prognostic and Predictive for Postoperative Adjuvant Chemotherapy Benefit in Patients with Gastric Cancer. Ann. Surg. 267 (2), 311–318. 10.1097/SLA.0000000000002058 27763900

[B72] Zhou J. Dudley M. E. Rosenberg S. A. Robbins P. F. (2005). Persistence of Multiple Tumor-specific T-Cell Clones Is Associated with Complete Tumor Regression in a Melanoma Patient Receiving Adoptive Cell Transfer Therapy. J. Immunother. 28 (1), 53–62. 10.1097/00002371-200501000-00007 15614045 PMC2175172

[B73] Zhu J. Wu G. Li Q. Gong H. Song J. Cao L. (2016). Overexpression of Suprabasin Is Associated with Proliferation and Tumorigenicity of Esophageal Squamous Cell Carcinoma. Sci. Rep. 6, 21549. 10.1038/srep21549 26899563 PMC4761926

