# Supplementary figures and images for: Role of Suprabasin in the Dedifferentiation of Follicular Epithelial Cell-Derived Thyroid Cancer and Identification of Related Immune Markers
Source: Front Genet. 2022 Feb 9;13:810681. doi: 10.3389/fgene.2022.810681 (PMC8865917; doi:10.3389/fgene.2022.810681)

group High Low

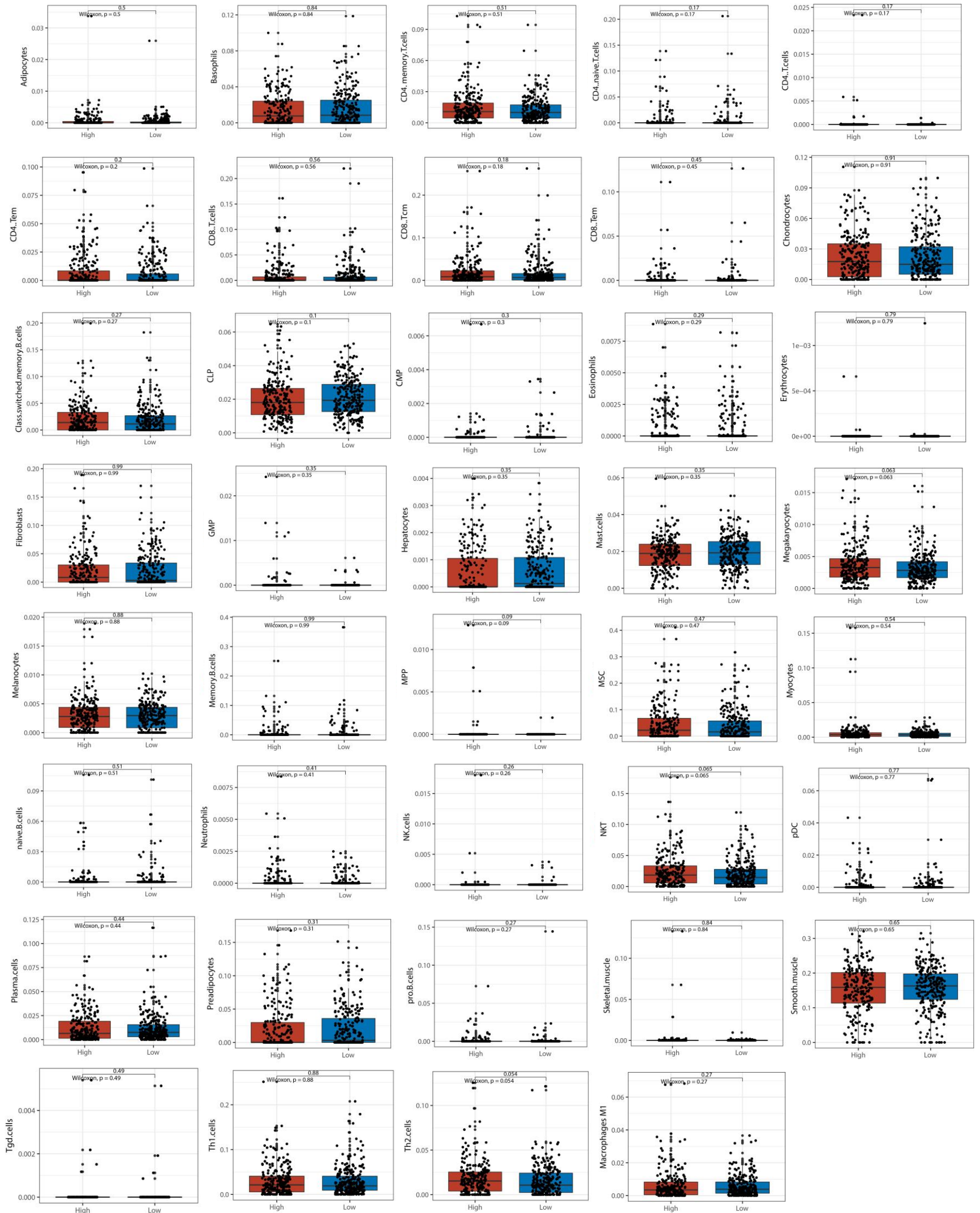

Supplement: Supplementary file 1 [file DataSheet1.ZIP › Fig.S2.pdf]

group    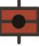 High    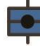 Low

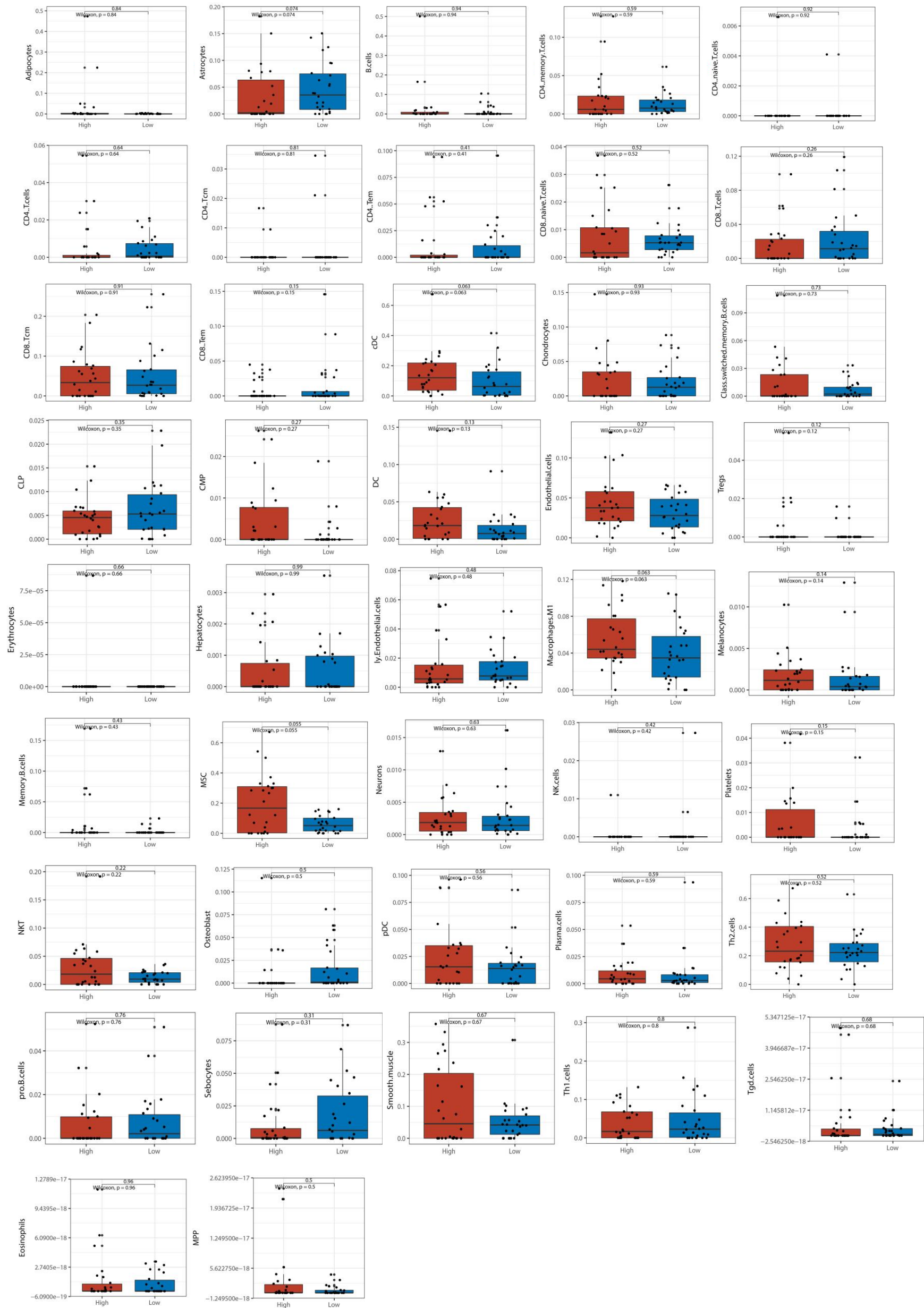

Supplement: Supplementary file 1 [file DataSheet1.ZIP › Fig.S3.pdf]

A

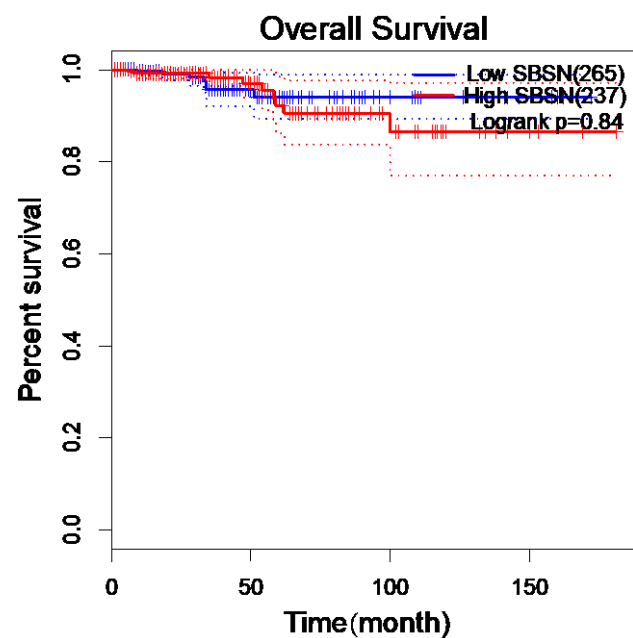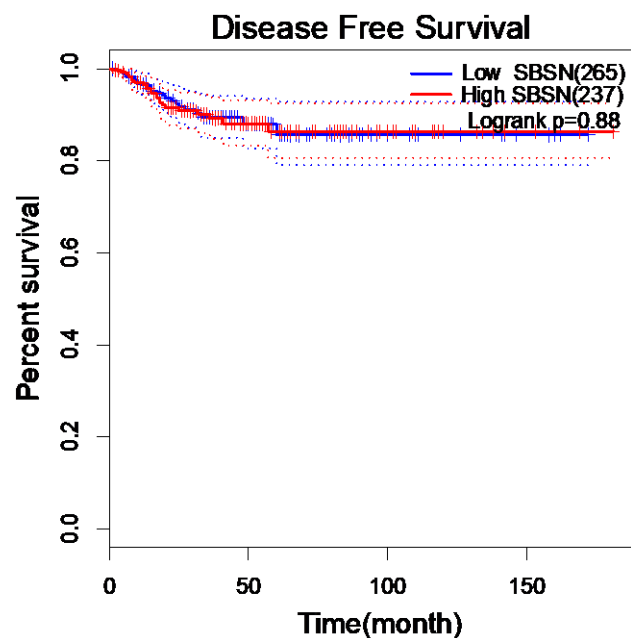

B

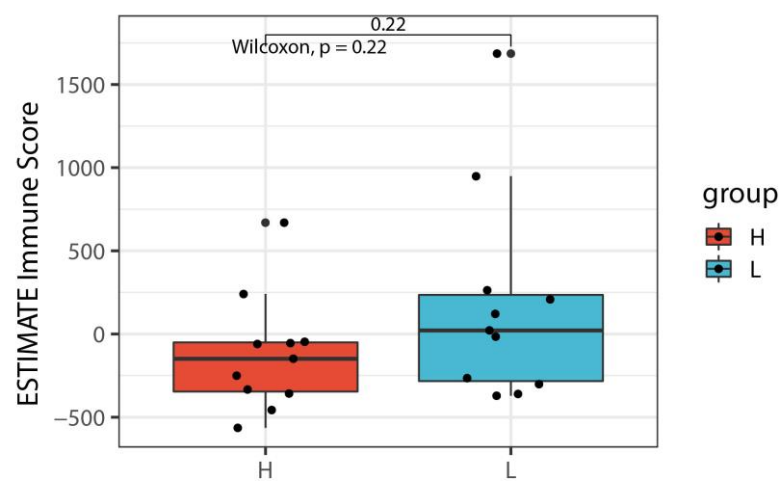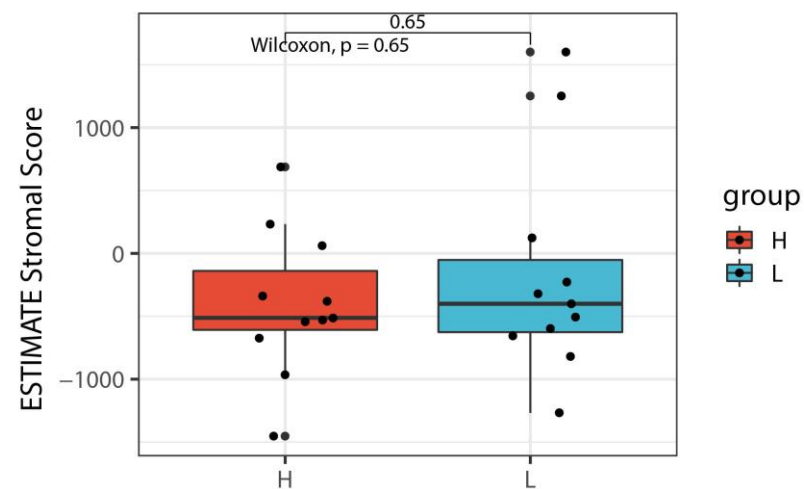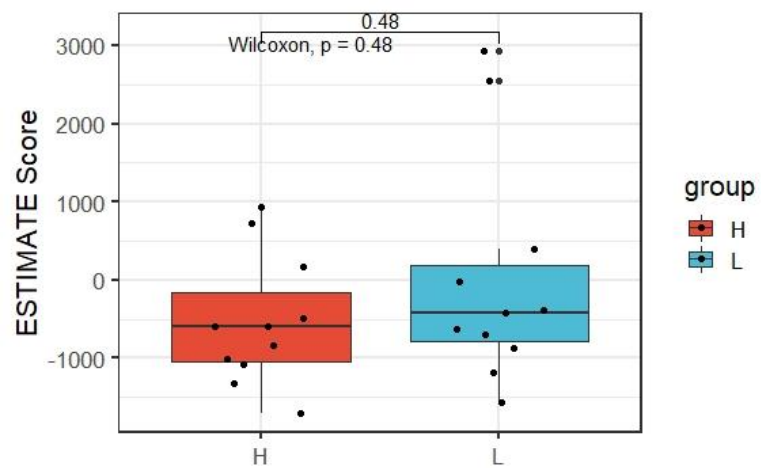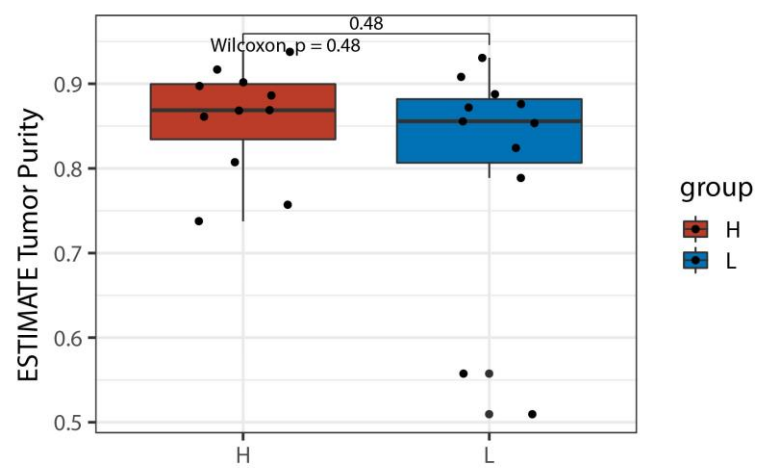

Supplement: Supplementary file 1 [file DataSheet1.ZIP › Fig.S1.pdf]
